# Genomic analysis reveals high intra-species diversity of *Shewanella algae*

**DOI:** 10.1099/mgen.0.000786

**Published:** 2022-02-10

**Authors:** Zhenzhou Huang, Keyi Yu, Songzhe Fu, Yue Xiao, Qiang Wei, Duochun Wang

**Affiliations:** ^1^​ National Institute for Communicable Disease Control and Prevention, Chinese Center for Disease Control and Prevention (China CDC), State Key Laboratory of Infectious Disease Prevention and Control, Beijing 102206, PR China; ^2^​ Center for Human Pathogenic Culture Collection, China CDC, Beijing 102206, PR China; ^3^​ Key Laboratory of Environment Controlled Aquaculture (KLECA), Ministry of Education, Dalian, PR China; ^4^​ College of Marine Science and Environment, Dalian Ocean University, Dalian, PR China

**Keywords:** *Shewanella algae*, diversity, genome characteristics, virulence-associated genes, drug resistance, environmental adaptability

## Abstract

*
Shewanella algae* is widely distributed in marine and freshwater habitats, and has been proved to be an emerging marine zoonotic and human pathogen. However, the genomic characteristics and pathogenicity of *Shewanella algae* are unclear. Here, the whole-genome features of 55 *S *. *algae* strains isolated from different sources were described. Pan-genome analysis yielded 2863 (19.4 %) genes shared among all strains. Functional annotation of the core genome showed that the main functions are focused on basic lifestyle such as metabolism and energy production. Meanwhile, the phylogenetic tree of the single nucleotide polymorphisms (SNPs) of core genome divided the 55 strains into three clades, with the majority of strains from China falling into the first two clades. As for the accessory genome, 167 genomic islands (GIs) and 65 phage-related elements were detected. The CRISPR-Cas system with a high degree of confidence was predicted in 23 strains. The GIs carried a suite of virulence genes and mobile genetic elements, while prophages contained several transposases and integrases. Horizontal genes transfer based on homology analysis indicated that these GIs and prophages were parts of major drivers for the evolution and the environmental adaptation of *S. algae *. In addition, a rich putative virulence-associated gene pool was found. Eight classes of antibiotic-associated resistance genes were detected, and the carriage rate of β-lactam resistance genes was 100 %. In conclusion, *S. algae* exhibits a high intra-species diversity in the aspects of population structure, virulence-associated genes and potential drug resistance, which is helpful for its evolution in pathogenesis and environmental adaptability.

## Data Summary

The sources and genomic sequences used throughout this study have been deposited in the National Centre for Biotechnology Information (NCBI), under the assembly accession numbers provided in Tables S1 and S2 (available in the online version of this article).

**Impact Statement**

*
S. algae* have become dominant species associated with human and aquatic livestock disease in the genus of *Shewanella *. Cases of bacteremia, sepsis, and infective endocarditis have also been reported. However, the evolution, biodiversity and pathogenic potential of *S. algae* are poorly understood. Through a comparative pan-genomic analysis of *S. algae* strains, we identified new pathogenic genomic islands, prophages, and virulence factors, suggesting that independent acquisition of these mobile genetic elements could play an important role in the evolution and virulence of *S. algae *. This study provides important insights into intra-species divergence and environmental adaptability underlying genomic sequences. The relationship between genome features and environmental adaptation strategies is an essential part for understanding the ecological functions of *S. algae* in the marine environments. The investigation of bacterial resistance shows that *S. algae* plays an important role as a reservoir of antibiotic resistance genes in the nature, which provides a scientific basis for surveillance and control of infectious diseases caused by *S. algae *.

## Introduction

*Shewanella* spp. are Gram-negative, oxidase-positive, H_2_S-producing, facultative anaerobe bacteria that are ubiquitously distributed in seafood, marine and freshwater environments, even in deep-sea and polar regions [1]. *Shewanella* spp. are characterized by prominent biodiversity, and they have evolved to develop complex lifestyles. The most outstanding ecological functions are bioremediation and application in the microbial fuel cells production [2, 3]. Various anaerobic respiration pathways and extracellular electron transfer are the metabolic features of the genus *Shewanella* [1, 4]. At the time of writing, this genus contains 74 recognized species (http://www.bacterio.net/shewanella.html).

Despite the beneficial roles, some species like *Shewanella algae* and *Shewanella putrefaciens* have been identified as opportunistic pathogens [5–7]. The proliferation and the clinical case reports of *Shewanella* spp. have raised the concern of microbiologists. Reported illnesses included skin and soft tissue infections, otitis media and bacteremia. By 2012, over 50 case reports of human infection caused by the genus *Shewanella* had been found in PubMed database [1]. Up to now, the cumulative number of *Shewanella* infections in literature has already exceeded 300. Recent advances in the taxonomy and phylogenetic relatedness of *Shewanella* spp. supported the concept that most human infections were caused by *S. algae* [5, 8].

Currently, multidrug resistance has been increasingly reported in the genus *Shewanella* [9–12]. Several antibiotic resistance determinants have been detected in this genus. Yousfi *et al*. discovered multidrug-resistant (MDR) plasmid in *S. xiamenensis* [10]. *Shewanella* spp. were resistant to different antibiotic classes, including β-lactams, quinolones and aminoglycosides. However, the genetic characterization of antimicrobial resistance in *Shewanella* spp. remains limited.

In recent years, the rapid development of high-throughput sequencing technology has brought opportunities for the genetic research of pathogens [13]. The improved sensitivity and accuracy of whole-genome sequencing technology are important for understanding the genome characteristics of pathogens. As increasing species of the genus *Shewanella* are sequenced, there are more studies focused on the whole genus rather than specific species. Fang *et al*. have constructed multilocus sequence analysis (MLSA) methods to accurately identify *Shewanella* strains at the species level [14]. Thorell *et al*. used the whole-genome sequencing to redefine *Shewanella* taxonomy [15]. As a predominant human pathogen in the genus *Shewanella *, *S. algae* is still largely unknown for its genomic characteristics, virulence factors and antimicrobial resistance. Therefore, in this study, we sequenced the genomes of 12 new *S. algae* strains and combined the genome sequences of 43 *S *. *algae* strains from public databases, to illustrate the intra-species hereditary and present a comprehensive analysis of *S. algae *.

## Methods

### Strain information

A total of 55 *S *. *algae* strains were used in this study, including 43 strains from public database (genomes with less than 200 contigs were selected) and 12 newly sequenced strains isolated from different sources in China (deposited in the Centre for Human Pathogenic Culture Collection, China CDC). These strains were isolated from China (*n*=38, 69.1 %), Tanzania (*n*=1, 1.8%), Korea (*n*=4, 7.3 %), Peru (*n*=1, 1.8 %), France (*n*=3, 5.5 %), Japan (*n*=3, 5.5 %), USA (*n*=2, 3.6 %), Argentina (*n*=1, 1.8 %), and unknown regions (*n*=2, 3.6 %), respectively. Also, according to the sources of isolation, there were 32 clinical strains and 23 environmental strains. It is worth noting that the *S. haliotis* strain JCM 14758 from GenBank was included, because *S. haliotis* has been recently identified as a synonym of *S. algae* by the previous research [14]. The 12 morphologically well-characterized strains in this study were initially identified by API 20E and 16S rRNA gene analysis. Detailed information of these strains was listed in Table S1.

### Genome sequencing

The genomic DNA was extracted by Wizard Genomic DNA Extraction Kit (Madison, WI, Promega, USA) following the manufacturer’s instructions. The HiSeq sequencer (Illumina HiSeq2000, San Diego, CA, USA) was used to perform 250 bp paired-end whole-genome sequencing with 150× coverage. FastQC (http://www.bioinformatics.babraham.ac.uk/projects/fastqc/) was used to evaluate the quality of the reads [16]. Low-quality reads were discarded if the quality scores of ≥3 consecutive bases were ≤Q30. SOAP *de novo* (version 2.04) was used to assemble the clean data of each strain [17]. After removing contigs with less than 500 bp, QUAST (version 5.0.1) software was applied to evaluate the quality of assembled genomes [18].

### Average nucleotide identity (ANI) analysis

ANI analysis was used to evaluate the evolutionary distance of bacteria at the genomic level based on a Perl script previously described [19]. ANI was calculated between the reference genome and the query genome, and then the ANI value representing the BLASTn matches was computed with the cutoff set as ≥30 % sequence identity and ≥70 % length coverage. The strains with the ANI value >95 % were considered as the same species [20].

### Pan-genome analysis

The genomes of 55 *S *. *algae* strains were collected together as a local database. Prokka (version 1.12) [21] was used to annotate genomes and produce standards-compliant *.gff output files for each sample. Roary pan-genome pipeline [22] with an identity cutoff i≥95 % was used. R package of pheatmap was used for the heatmap of the presence or absence of all these pan genes.

### Core genome comparison

Coding sequences prediction was carried out using Prodigal (version 2.6.3) [23]. Then a non-redundant homologous gene set was computed for 55 strains using CD-HIT [24]. Next, BLAST+ was used to search the homologous genes in the non-redundant homologous gene set of each strain with the cutoff value of ≥90 % sequence identity and ≥60 % length coverage. If a homologous gene found in the non-redundant homologous gene set had just one copy in each strain and existed in all strains, it was considered as a core gene. The core genes were then aligned, and Gubbins (http://github.com/sanger-pathogens/gubbins) was used as a recombination-removal tool to reorganize the core genome. PhyML (version 3.1) [25] was used to construct the phylogenetic trees by maximum-likelihood method based on all these core SNPs (bootstrap replications, 1000). The strain RQs-106 with complete genome was selected as a reference to explore the number of SNPs for other strains. Population structure was defined using FastBaps (https://github.com/gtonkinhill/fastbaps) by a fast hierarchical Bayesian analysis [26].

### Genomic islands (GIs), prophages, plasmids, CRISPR and unique genes analysis

Accessory genes were analysed using three online databases with default parameters. IslandViewer (http://www.pathogenomics.sfu.ca/islandviewer/) is a widely-used webserver for the prediction and interactive visualization of GIs (regions with probable horizontal origin). It integrates with IslandPath-DIMOB, SIGI-HMM and IslandPick to make accurate and complementary GI predictions [27]. PHASTER (PHAge Search Tool Enhanced Release) (http://phaster.ca) was used for rapid identification and annotation of prophage sequences in bacterial genomes and plasmids. An online search was performed against a custom prophage/phage database containing protein sequences from NCBI phage database developed by Srividhya *et al*. [28]. Plasmid replicons were identified online (http://www.genomicepidemiology.org/). CRISPRCasFinder (https://crisprcas.i2bc.paris-saclay.fr/CrisprCasFinder) was used for the detection of CRISPRs (clustered regularly interspaced short palindromic repeats) and Cas (CRISPR-associated proteins) genes in the submitted sequence data. A blast comparison was performed against NCBI-NR database using the protein sequences of strain-unique genes with default parameters. The R package of plotrix was used to form the flower plot of unique genes.

### Virulence-related genes analysis

The Virulence Factor Database (VFDB) [29] was used to predict virulence-related genes by blast+. The parameters included an E-value of 1e-5, a minimum identity and coverage of 50% and 70 %, respectively.

### Antimicrobial resistance genes analysis

Potential antimicrobial resistance genes were predicted by Comprehensive Antibiotic Research Database (CARD) (http://arpcard.mcmaster.ca). The parameters of BLAST+ were an E-value of 1e-5, the percentage of sequence identity ≥80 % and the percentage of length coverage ≥80 %.

### Antimicrobial susceptibility testing

The Antimicrobial Susceptibility Testing (AST) panel for aerobic Gram-negative bacilli (Shanghai Fosun Long March Medical Science Co., Ltd., China) was used to perform the antimicrobial susceptibility testing by the micro-broth dilution method. Each antibiotic has a concentration gradient on the drug sensitive plate. The bacteria suspension was added to the plate, and then after an incubation period of 18 to 20 h, the results were interpreted. The minimal inhibitory concentration (MIC) values were obtained. There is no CLSI breakpoints for *Shewanella,* and the results of susceptible (S), intermediate (I), and drug resistance (R) were interpreted according to the *Enterobacteriaceae* standards of the American Committee for Clinical Laboratory Standardization (CLSI) [30]. *Escherichia coli* ATCC 25922 was used as a control for chloramphenicol, tetracyclines, sulfonamides, and trimethoprim-sulfamethoxazole.

## Results

### General features of *S. algae* genomes

**Fig. 1.**
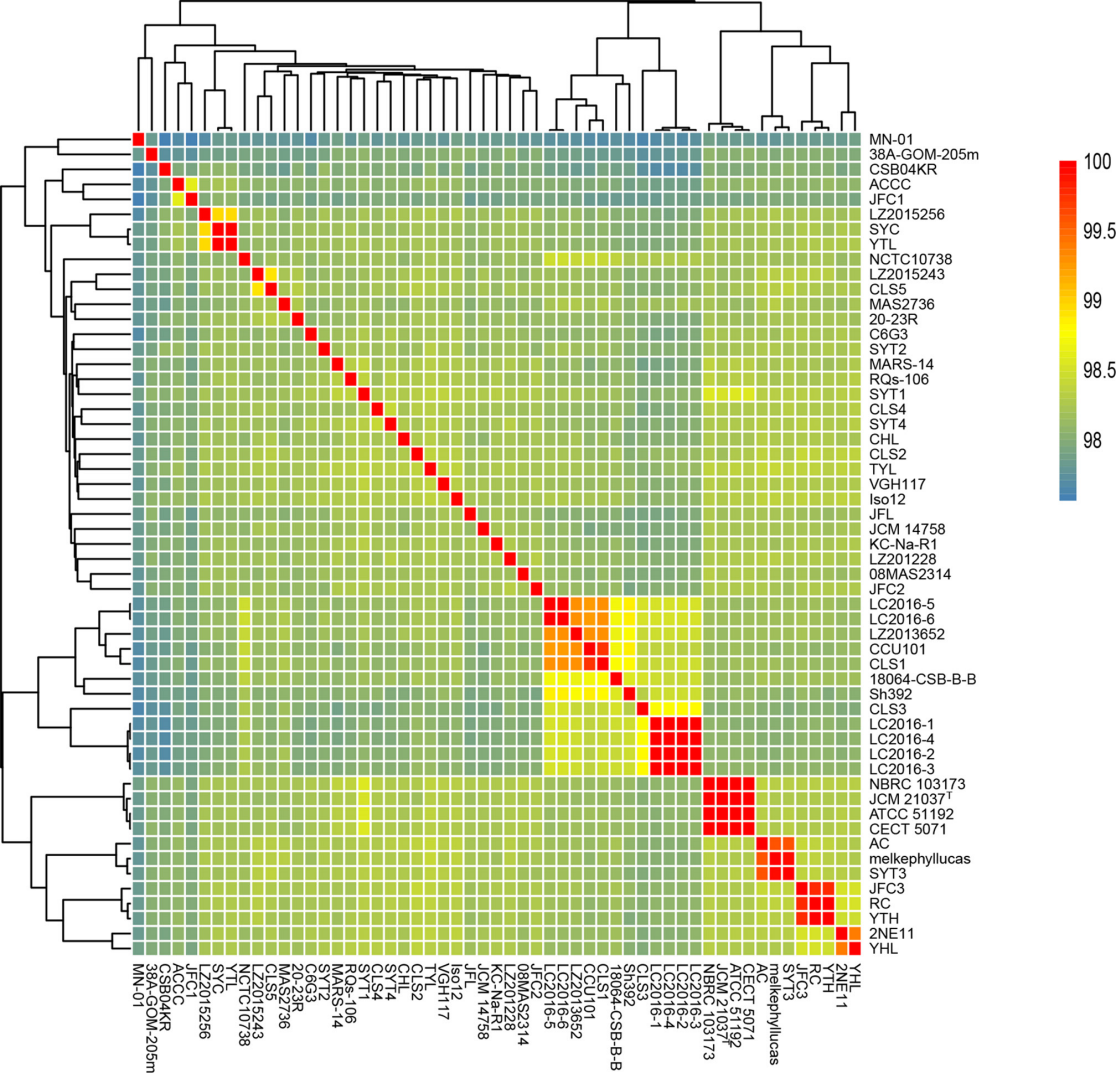
Average nucleotide identity (ANI) values for the whole genomic sequences of the 55 *S *. *algae* strains.

The genome sizes of the 55 strains ranged from 4.60 Mb (strain CLS3) to 5.20 Mb (strain KC-Na-R1), and the G+C content ranged from 52.5 mol% to 53.1 mol%. The general information of the 55 genomes was summarized in Table S2. Based on the matrix of ANI values, the dendrograms were drawn (Fig. 1). The ANI values of the 55 strains ranged from 97.7–99.9 %, providing accurate identification at the species level. The relatively high ANI values were found among three groups (LC2016-1/LC2016-2/LC2016-3/LC2016-4; NBRC 103673/JCM 21037^T^/ATCC 51192/CECT 5071; JFC3/RC/YTH).

### Pan genome analysis

**Fig. 2.**
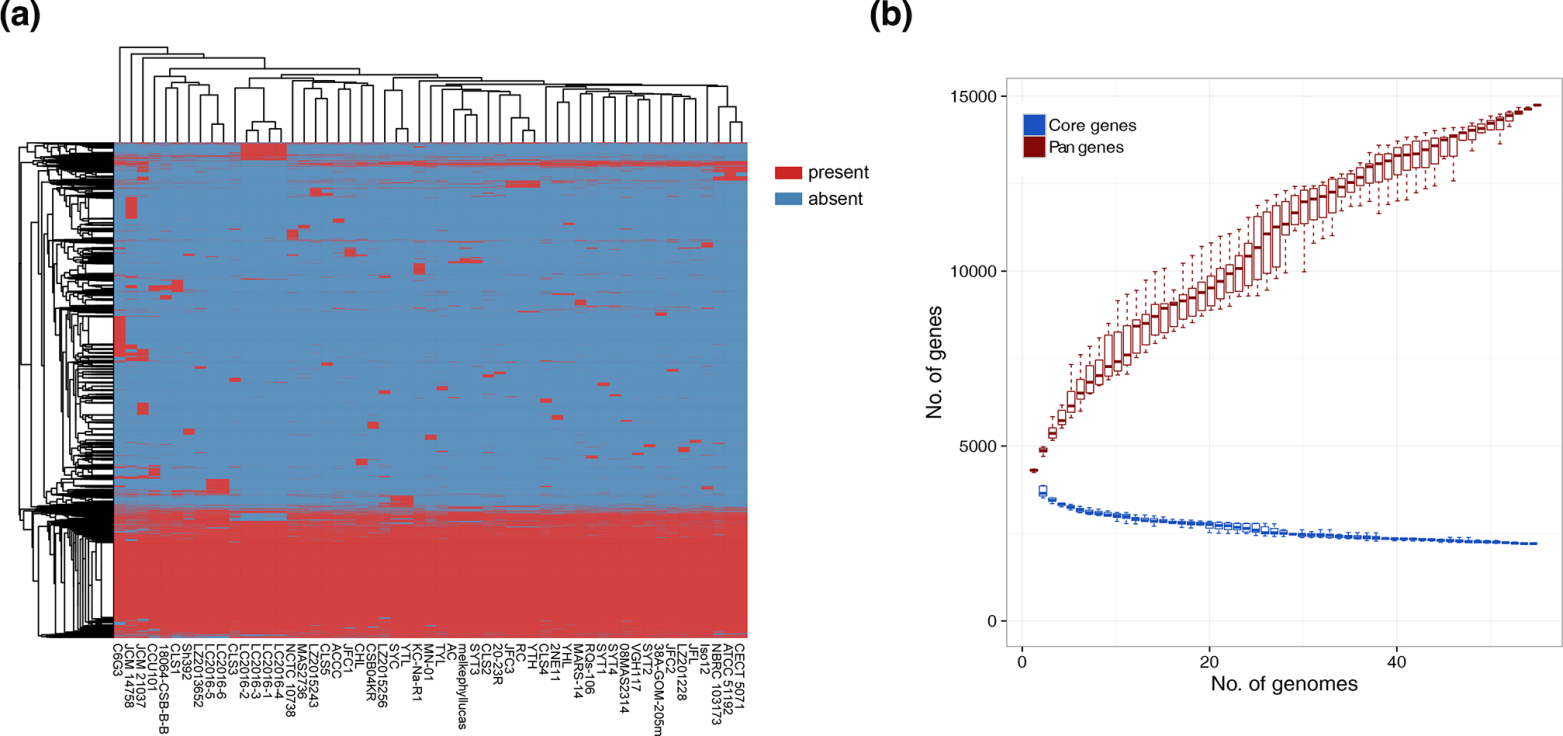
Pan genome analysis of 55 *S *. *algae* strains and dilution curves of core/pan genes. (a): The heatmap based on pan genome sequences. On the top was the cluster of strains, and on the left was the cluster of pan genes. The red colour represented the presence of genes, while the blue colour represented the absence. (b): The dilution curves of core/pan genes.

A total of 14780 genes, which represented the pan-genome of 55 *S *. *algae* strains, were found, with the CDSs ranging from 4105 (strain CLS3) to 5694 (strain C6G3) (Fig. 2a). Among them, 2863 (19.4 %) genes were shared by all strains and comprised the core genome. The COG functional annotation of the core genome focused on genes related to the basic lifestyle such as metabolism and energy production. The length of the core genome was 1 506 597 bp, and a total of 120 559 SNPs were detected. Taking the complete genome of strain 2NE11 as a reference, the number of SNPs ranged from 7496 (strain YHL) to 25 395 (strain MN-01), with the average number of 21 162. The dilution curve showed that the number of novel gene families in the pan-genome constantly increased when more genomes were considered, while the number of conserved genes constituting the core genome gradually stabilized (Fig. 2b).

### Population structure analysis

**Fig. 3.**
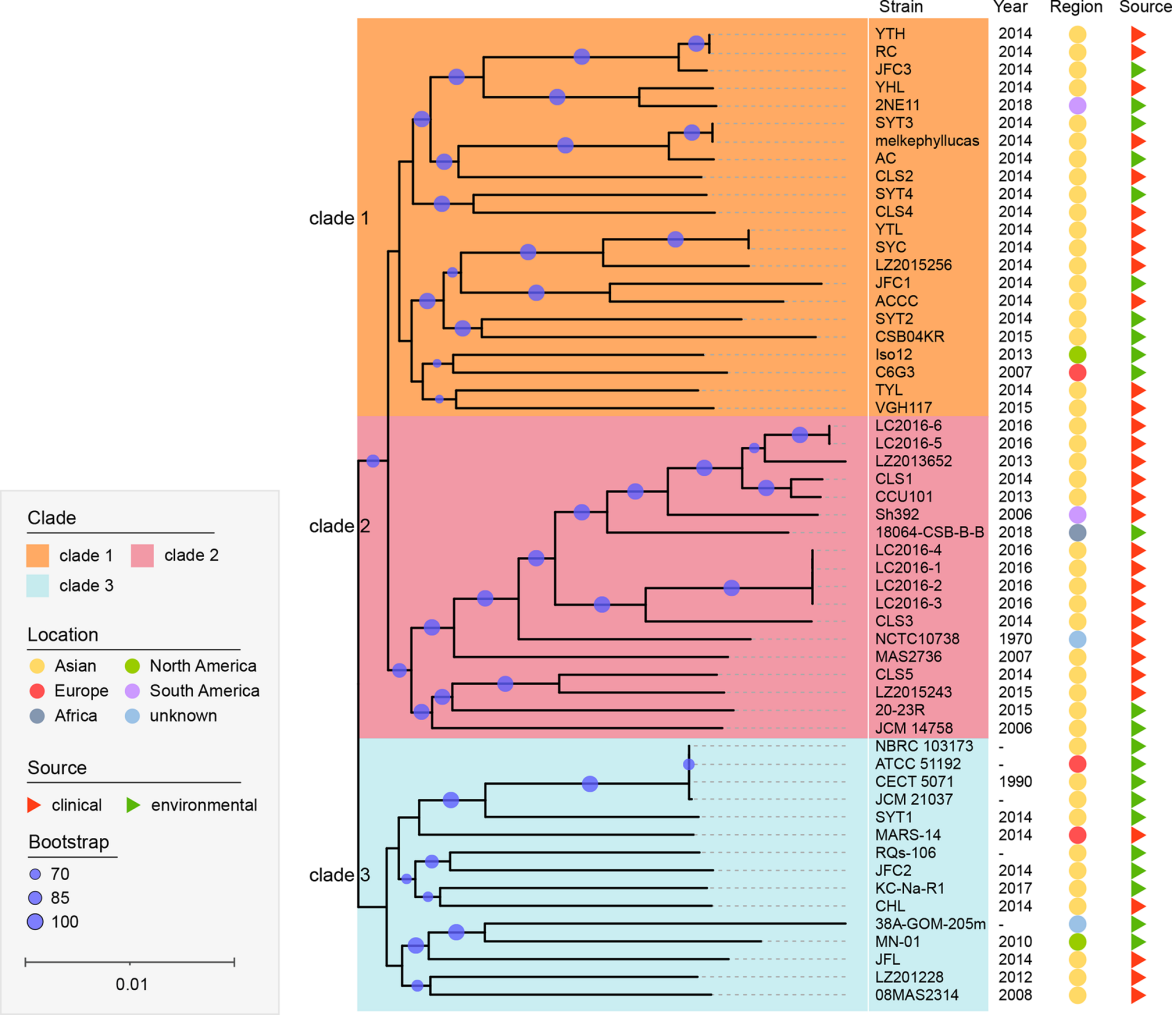
Phylogenetic tree of core genome sequences by maximum-likelihood method. The strains were divided into six clades. The isolation year, regions and sources were shown on the right. Regions were circled in different colours and sources are triangulated (green for environmental sources and red for clinical sources). The robustness of tree topologies was evaluated with 1000 bootstrap replications. The scale represented a nucleotide substitution rate of 0.01 for each site.

A robust core genome sequences based ML-tree was inferred to investigate the phylogenetic relationship of the 55 *S *. *algae* strains (Fig. 3). Three clades were identified according to the hierarchical Bayesian analysis by FastBaps. Clade 1 contained the largest number of strains (*n*=22), followed by clade 2 (*n*=18) and clade 3 (*n*=15). Among the 38 strains isolated from China, 31 strains were clustered in clade 1 and clade 2. Most strains derived from clinical patients appeared to be clustered in clade 2. The distribution of pairwise SNP distances (Fig. S1) showed that most isolates in clade 2 had a closer pairwise evolutionary relationship than those in clade 1 and clade 3.

### Inter-species relationship between *S. algae* and other phylogenetically closed *Shewanella* spp

**Fig. 4.**
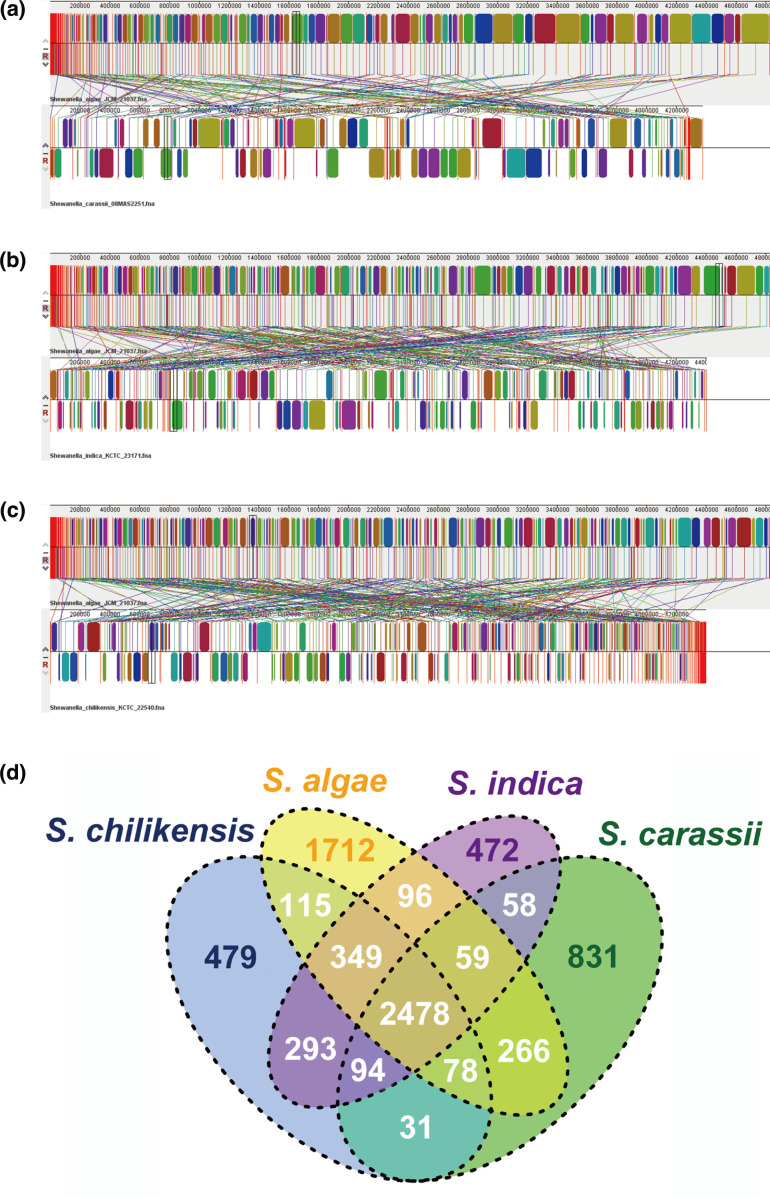
The collinearity and the phylogeny among *Shewanella* spp. (a): General comparisons between *S. algae* JCM 21037^T^ and *S. carassii* 08MAS2251^T^ presented by Mauve software. (b): General comparisons between *S. algae* JCM 21037^T^ and *S. indica* KCTC 23171^T^ presented by Mauve software. (c): General comparisons between *S. algae* JCM 21037^T^ and *S. chilikensis* KCTC 22540^T^ presented by Mauve software. The Mauve parameter settings were default. (d): Venn diagram of the shared and unique genes found in *S. algae* JCM 21037^T^ and other three *Shewanella* genomes.

Comparative genomic analysis of *S. algae* JCM 21037^T^ and three strains of other *Shewanella* spp. with the closed genetic relationships (* S. carassii* 08MAS2251^T^, *S. chilikensis* KCTC 22540^T^ and *S. indica* KCTC 23171^T^) was performed, according to the report of Thorell *et al*. [15]. We preliminarily evaluated the rearrangement and duplication events within the genomes through genome collinearity comparison (Fig. 4a–c). The three-genome alignments yielded 61, 65, and 68 Mauve blocks, respectively. The overall chromosome structures differed considerably among these strains. The lack of conservation appeared to be the result of a large inversion and multiple expansions of repetitive regions between the genomes of strains. Also, the Venn diagram of the shared and unique genes demonstrated that *S. algae* JCM 21037^T^, *S. carassii* 08MAS2251^T^, *S. chilikensis* KCTC 22540^T^ and *S. indica* KCTC 23171^T^ strains shared 2478 core genes (Fig. 4d), and these four strains harboured 1712, 831, 479, and 472 unique genes, respectively. An additional gene set, ranging from 31 to 293 genes, was shared by any two strains, while the number of genes that were shared by any three strains varied from 59 to 349.

### GIs, prophages, plasmids, CRISPR and unique genes analysis

One hundred and sixty-seven GIs from six complete genomes of *S. algae* strains (2NE11: *n*=37, RQs-106: *n*=37, VGH117: *n*=25, 18064-CSB-B-B: *n*=24, CCU101: *n*=14, KC-Na-R1: *n*=30) were detected. The detected genes were related to virulence, metabolism, resistance, structure, fitness factors, modification-restriction systems, transport and toxins. GIs related to mobile element protein, integrase, transposase, acetyltransferase and transcriptional regulator were found in all six strains. Multidrug resistance transporter and heat shock protein were found in both RQs-106 and VGH117.

Sixty-five different prophage-related elements were detected, in which four genomes (strains KC-Na-R1, RQs-106, JFL and melkephyllucas) had intact prophages. Specifically, 40 (61.5 %) prophage-related sequences with the length between 4.4 and 42 kb were unique in 24 strains (Table S3), while five prophage-related sequences were shared by *S. algae* strains (Table S4). The detection of the presence of fitness factors encoded inside these sequences showed that 19 sequences carried transposase-like elements and 15 sequences contained integrase-like elements linked to horizontal gene transfer.

Plasmids were found in six strains, in which four strains were isolated from clinical specimens and the remaining two were sourced from environment. Three types of plasmid replicons were predicted. The information about the antimicrobial resistance genes and the functions of products encoded by the virulence-associated genes were described. The strains 18064-CSB-B-B and CCU101 appeared to carry resistance-transfer factors (RTFs). For complete genomes (18064-CSB-B-B and CCU101), the plasmid lengths were defined, otherwise the plasmid replicons were just localized. The detailed information was shown in Table S5.

Credible CRISPR-Cas system (evidence level=4) was predicted in the entire *S. algae* genomes. Two Cas types were detected, namely Cas-TypeIE for strains NCTC 10738, JFC2, JFL, SYT2 and CHL, and Cas-TypeIF for the others. The number of spacer sequences contained in CRISPR varied greatly among different strains. Specifically, strain C6G3 only harboured CRISPR sequences but not Cas clusters. The detailed information was shown in Table S6.

**Fig. 5.**
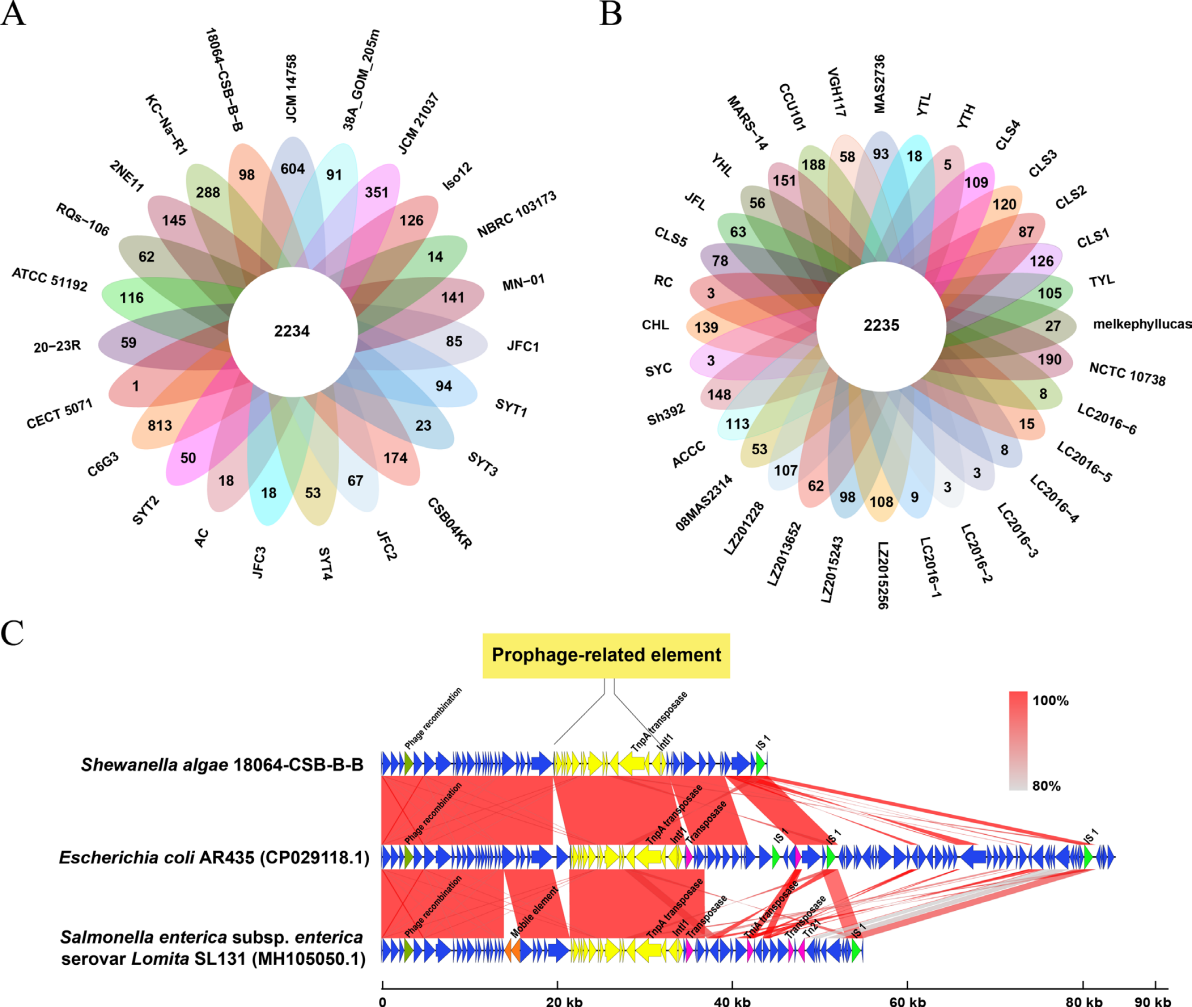
The distribution of unique genes in environmental vs. clinical isolates and the gene clusters composition of prophage-related element. (a): The unique genes distribution of environmental strains. (b): The unique genes distribution of clinical strains. (c): The comparison of nucleotide similarity of the prophage-related element in *S. algae* 18064-CSB-BB and the homologs in *E. coli* and *Salmonella enterica* strains. The prophage-related CDSs were represented in yellow, while the insert sequence 1 (IS 1) in emerald green, the transposons in pink, the phage recombination associated CDSs in dark green, the other unnamed mobile elements in orange and the gene clusters in blue.

A large number of CDSs were annotated as hypothetical proteins. The number of unique genes in the 55 *S *. *algae* strains ranged from one to 813 (Fig. 5a, b). The strain C6G3, isolated from French seabed sediments in 2007, was found to contain the highest number of unique genes (813), while the strain CECT 5071 contained the fewest (one). The average number of unique genes was 75 in the clinical strains (Fig. 5a), and 106 in the environmental strains (Fig. 5b) (Wilcoxon rank sum test, no significant statistical difference, *P*>0.05).

Four GIs related to cross-species transmission were identified in the 55 *S *. *algae* genomes. Two conjugative transfer-related GIs detected in strain 2NE11 shared 84.47 and 95.90% sequence identity with *Halomonas meridiana* Eplume2 and *Alcanivorax* N3-2A respectively. Two GIs detected in strains RQs-106 and CCU101 shared 93.35 and 97.15% sequence identity with *Aeromonas hydrophila* OnP3.1 and *Marinobacter hydrocarbonoclasticus* VT8 respectively (Table S7). The prophage-related element, playing the role of transposase, was detected in strain 18064-CSB-B-B isolated from poultry stool. Interestingly, this prophage-related element was found to be very similar to those in *E. coli* AR435 and *Salmonella* SL131 by blast homology comparison (Fig. 5c). Large quantities of insert sequences (IS) and transposases were predicted and annotated in the upstream and downstream sequences of these mobile genetic elements (MGEs), which may contribute to the genetic diversity of the accessory genome.

### Prediction of virulence-associated genes

**Fig. 6.**
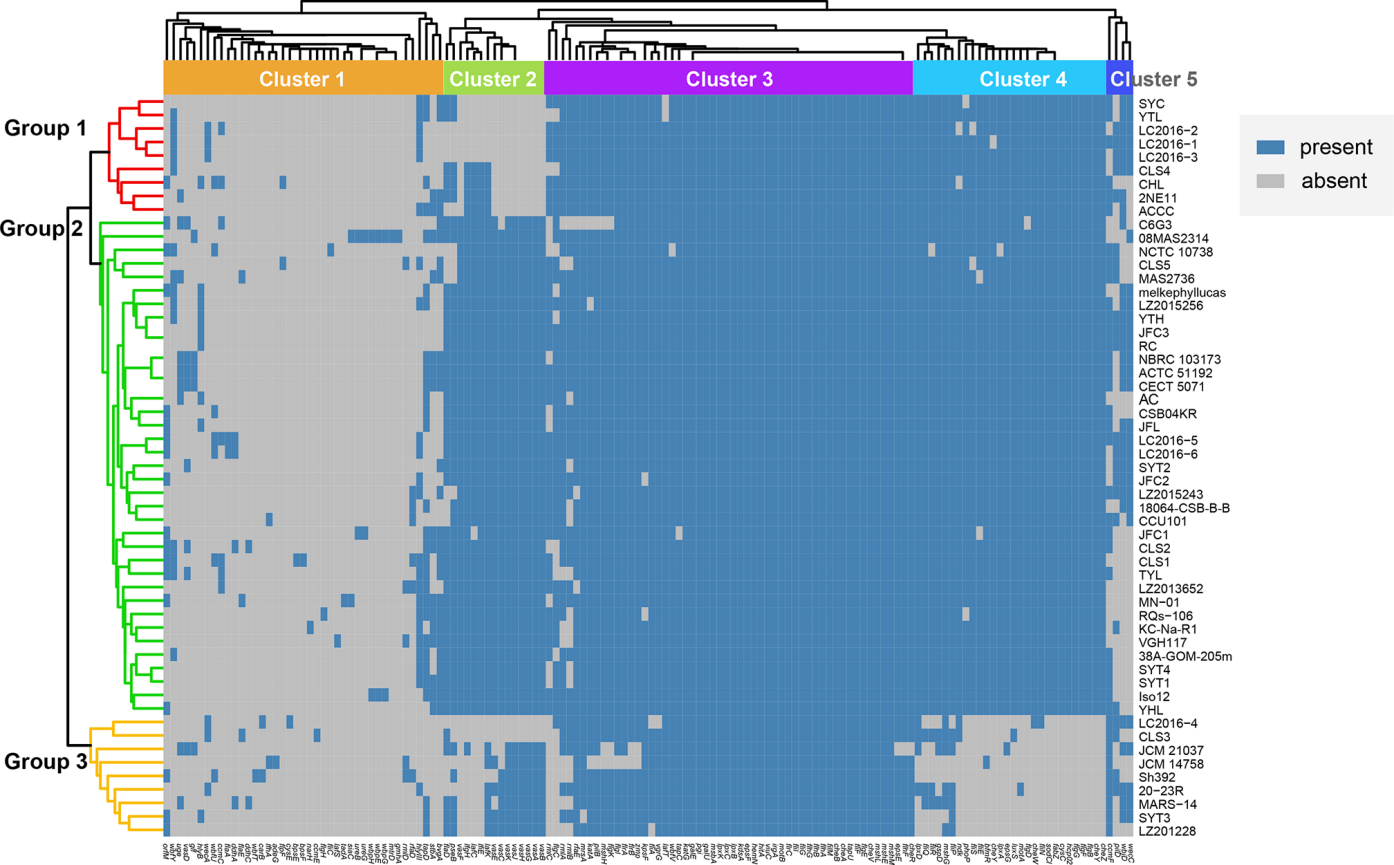
The heatmap of the virulence genes in 55 *S *. *algae* strains. On the left, the *S. algae* strains were divided into three groups (Group 1, Group 2 and Group 3) according to the clustering situation. On the top was the clusters of virulence-related genes, which were marked as Cluster 1 to Cluster 5, respectively. The blue colour indicated the presence of genes while the grey colour indicated the absence.

In the VFDB database, due to the lack of a virulence factor library related to *Shewanella* genus, we used *Vibrio* species as a reference for the virulence gene analysis because the phylogenetic relationship of *Vibrio* was closed to *Shewanella *. A putative virulence-associated genes pool showed great diversity, consisting of 19 classes, 59 virulence factors and 142 potential virulence genes (Table 1). The virulence-associated genes can be divided into five clusters, and the 55 *S *. *algae* strains were classified into three groups (Fig. 6). The results showed that 55 to 107 known or putative virulence genes were identified in the genome of each strain. The strains of different groups manifested specific virulence gene patterns. Most strains carried potential virulence genes related to secretion system, iron uptake and adherence, but rarely carried genes of Cluster 1, which could encode toxin, endotoxin, antiphagocytosis, acid resistance, biofilm formation and nutritional virulence. Strains from Group 1 mainly contained genes of Cluster 4 and 5, while strains from Group 2 mainly contained genes of Cluster 2, 4 and 5. Nearly all strains in Group 2 carried virulence-related genes in Cluster 3 with the main functions of secretion system, adherence and glycosylation system. The gene *vasF* related to VAS type VI secretion system was specific for strains in Group 2. Although strains from Group 3 mainly carried genes of Cluster 2 and 5, most strains in this group lacked a portion of potential virulence gene in Cluster 4 related to flagella, two component system, EPS type II secretion system, autoinducer-related genes and VAS effector proteins. On the whole, strains in Group 3 contained relatively the lowest number of virulence-associated genes, while strains in Group 2 contained the highest number.

**Table 1.** The classification of virulence related factors of *S. algae* species against VFDB

Cluster	VF-class	Virulence factor	Functions of productions
C1	Acid resistance	Urease	Protein biosynthesis
C1	Adherence	Accessory colonization factor, Mannose-sensitive hemagglutinin4, Type IV pilus, Flagella4, Flp type IV pili, LPS O-antigen, Tap type IV pili, *tad* locus	Flagellum assembly, pilus assembly, protein secretion, signals transduction, translocation, hydrolase
C1	Anaerobic respiration	Nitrate reductase	Catalytic activity
C1	Antiphagocytosis	Capsular polysaccharide	LPS biosynthesis and metabolism, catalytic activity
C1	Biofilm formation	AdeFGH efflux pump/transport autoinducer	Catalytic activity
C1	Chemotaxis and motility	Flagella	Flagellum biogenesis, motor activity, pathogenesis
C1	Efflux pump	AcrAB, MtrCDE	Efflux
C1	Toxin	Hemolysin III, Phytotoxin phaseolotoxin, Endotoxin	Signalling receptor, protein kinase activity
C1	Immune evasion	Capsule, LPS, Exopolysaccharide	Metabolism, stationary phase survival, catalytic activity, biosynthesis
C1	Nutritional virulence	Biotin metabolism, Cysteine acquisition, Pyrimidine biosynthesis	Biotin metabolism, chemotaxis signal transduction, signals transmission
C2	Adherence	Accessory colonization factor, Mannose-sensitive hemagglutinin4, Type IV pilus, Flagella4, Flp type IV pili, LPS O-antigen, Tap type IV pili, *tad* locus	Flagellum assembly, pilus assembly, protein secretion, signals transduction, translocation, hydrolase
C2	Chemotaxis and motility	Flagella	Flagellum biogenesis, motor activity, pathogenesis
C2	Secretion system	EPS type II secretion system, VAS type VI secretion system3, T4SS effectors	Protein secretion and transport, virulence, chaperone
C2	Glycosylation system	O linked flagellar glycosylation	Catalytic activity
C3	Adherence	Accessory colonization factor, Mannose-sensitive hemagglutinin4, Type IV pilus, Flagella4, Flp type IV pili, LPS O-antigen, Tap type IV pili, *tad* locus	Flagellum assembly, pilus assembly, protein secretion, signals transduction, translocation, hydrolase
C3	Antiphagocytosis	Capsular polysaccharide	LPS biosynthesis and metabolism, catalytic activity
C3	Chemotaxis and motility	Flagella	Flagellum biogenesis, motor activity, pathogenesis
C3	Immune evasion	Capsule, LPS, Exopolysaccharide	Metabolism, stationary phase survival, catalytic activity, biosynthesis
C3	Iron uptake	Enterobactin receptors, Periplasmic binding protein-dependent ABC transport systems, Cytochrome c maturation locus, Haemophilus iron transport locus, biosynthesis	Transcription activation, signalling receptor, transport, catalase activity
C3	Endotoxin	Lipooligosaccharide (LOS) (Haemophilus)	Catalytic activity
C3	Protease	Zn^2+^ metallophrotease	Endoprotease
C3	Stress adaptation	Catalase, *SodCI*	Catalytic activity, destroys radicals
C4	Adherence	Accessory colonization factor, Mannose-sensitive hemagglutinin4, Type IV pilus, Flagella4, Flp type IV pili, LPS O-antigen, Tap type IV pili, *tad* locus	Flagellum assembly, pilus assembly, protein secretion, signals transduction, translocation, hydrolase
C4	Chemotaxis and motility	Flagella	Flagellum biogenesis, motor activity, pathogenesis
C4	Endotoxin	LOS (Haemophilus)	Endotoxin
C4	Immune evasion	Capsule, LPS, Exopolysaccharide	Metabolism, stationary phase survival, catalytic activity, biosynthesis
C4	Phagosome arresting	Nucleoside diphosphate kinase	Nucleoside triphosphates synthesis
C4	Quorum sensing	Autoinducer-2, PhoPQ, Two component system	Autoinducer synthesis, regulation of transcription
C4	Regulation	Two component system	Signal transduction
C4	Stress adaptation	Catalase, *SodCI*	Catalytic activity, destroys radicals
C4	Toxin	Hemolysin III, Phytotoxin phaseolotoxin, Endotoxin	Signalling receptor, protein kinase activity
C4	Secretion system	EPS type II secretion system, VAS type VI secretion system3, T4SS effectors	Protein secretion and transport, virulence, chaperone
C5	Iron uptake	Enterobactin receptors, Periplasmic binding protein-dependent ABC transport systems, Cytochrome c maturation locus, Haemophilus iron transport locus, biosynthesis	Transcription activation, signalling receptor, transport, catalase activity
C5	Adherence	Accessory colonization factor, Mannose-sensitive hemagglutinin4, Type IV pilus, Flagella4, Flp type IV pili, LPS O-antigen, Tap type IV pili, *tad* locus	Flagellum assembly, pilus assembly, protein secretion, signals transduction, translocation, hydrolase
C5	Antiphagocytosis	Capsular polysaccharide	LPS biosynthesis and metabolism, catalytic activity

### *S. algae* as a reservoir and a vehicle of potential antimicrobial resistance

We found genes associated with the resistance to eight antibiotics in the *S. algae* genomes (Table 2). The genes resisted to β-lactams (*bla*_OXA_, *bla*_CTX_, *bla*_CMY_, *bla*_TEM_, *bla*_VEB_), aminoglycosides (*armA*, *aph(3'')-Ib*, *aph(6)-Id*, *ant(2'')-Ia*, *aadA2*), quinolones (*qnrA*), phenicols (*cml*, *catA1*, *floR*), macrolides (*erm*), sulfonamides (*dfr*, *sul*), tetracyclines (*tet*), lincosamides (*vga*) were detected by BLAST+ against the database. The *bla*_OXA_ gene was located in all strains with the major genotype of *bla*_OXA-SHE_ (81.8%). Thirty-six strains carried *qnr* with the most common genotype of *qnrA3*. The environmental strain KC-Na-R1 carried the highest number of antimicrobial resistance genes, which indicated a capacity to resist multiple antibiotics. The genetic environments of identified resistance genes had been analysed. Twenty-two strains had mobile genetic elements (MGEs) in the upstream and downstream 10 kb sequences of resistance genes. The resistance genes possessed by strain KC-Na-R1 had the most abundant MGEs in its genetic environment. The detailed information was shown in the Table S8.

**Table 2.** Distribution and positive rate of antimicrobial resistance genes carried by 55 *S *. *algae* strains

Category	Genotype	no. of strains	Positive rate (%)
β-lactam	*bla*_OXA-SHE_, *bla*_OXA-10_, *bla*_OXA-55*,*_ *bla*_CTX-M-15_, *bla*_CMY-2_, *bla*_TEM-2_, *bla*_VEB-1_	55	100
Quinolones	*qnrA1, qnrA3, qnrA5, qnrA7*	36	65.5
Aminoglycosides	*armA*, *aph(3'')-Ib*, *aph(6)-Id*, *ant(2'')-Ia*, *aadA1*, *aadA2*	12	21.8
Sulfonamides	*dfrA12*, *dfrA27, sul1, sul2*	12	21.8
Amide alcohols	*cmlA1*, *catA1*, *floR*	8	14.5
Lincosamides	*vga(A*)	1	1.8
Tetracyclines	*tet(A*)	1	1.8
Macrolides	*erm(42*)	1	1.8

### Antibiotic resistance screening of *S. algae* strains

The antimicrobial susceptibility of 12 clinical strains was shown in Table S9. All strains were resistant to cefazolin, but sensitive to minocycline, amikacin, cefepime, meropenem, doxycycline, minocycline, gentamicin, kanamycin, streptomycin. Also, these strains were sensitive to four kinds of aminoglycosides (including gentamicin, amikacin, kanamycin, and streptomycin). Six strains isolated from patients of Laizhou, China in 2015 were multi-drug resistant (insensitive to three or more types of antibiotics).

## Discussion

Our study firstly provided a finer-scale comparison of *S. algae* at the intra-species level by sequencing 12 new *S. algae* strains and performed pan-, core- and accessory-genome analysis together with another 43 *S *. *algae* strains available in GenBank.

Pan-genome analysis explored the gene pool, unique genes and functional information underlying bacterial diversification. The number of accessory genes was 5.2 times that of core genes, which demonstrated that *S. algae* has great diversity in terms of genomic characteristics. The phylogenetic tree based on core genome demonstrated that the 55 *S *. *algae* strains were clustered into three separate clades, with most strains in China in clade 1 and 2. Compared with the report by Fang *et al*. [14], the phylogeny of *S. algae* in our study was consistent with the topological structure and evolutionary relationship of the phylogenetic tree constructed by MLSA method. When comparing the type strain of *S. algae* with those of other phylogenetically closed *Shewanella* species based on genome alignments, we observed that there were a large number of rearrangement and duplication events within different *Shewanella* species. The COG function annotation showed that the unique genes of *S. algae* were mainly related to signal transduction and transcription mechanisms, which may also indicate that accessory genes, especially the unique genes acquired from different environments, could be important for *S. algae* strains to increase their survival ability (Fig. S2).

Currently, the pathogenic mechanism of *S. algae* is not clear. Wu *et al*. found homologous genes of *hlyA*, *hlyB*, *hlyD* and *tolC* in the genome of *S. algae* STY3, which encoded hemolysin and transport functions, and speculated that the hemolytic activity may be an important virulence factor of *S. algae* [31]. Gallacher *et al*. confirmed that *S. algae* begins to produce tetrodotoxin after 12 h of culture [32]. Also, TTX was detected in *S. algae* strains isolated from food poisoning specimens by Wang *et al*., which suggests the possibility of TTX as a pathogenic substance of *S. algae* [33]. Alazea M. Tamez *et al*. have discovered and proposed candidate virulence factors of *S. algae *, including hemolysin, the type VI secretion system (T6SS), microbial collagenase, DNase, type IV pili, curli, twin-arginine translocation system, ClpP and urease [34].

In order to further explore the pathogenic characteristics of *S. algae *, 142 potential virulence-associated genes were extracted against the VFDB online database, showing a rich diversity of virulence-associated factors. There were significant differences in the virulence genes carried by strains in different groups (Fig. 6). Thirty-four core virulence-associated genes were found in all 55 strains. The presence of *hlyB* was only found in eight strains, and the complete RTX family that encodes a hemolysin failed to be predicted, which may explain why the function of hemolysis was irregular and difficult to be detected by Janda *et al*. [5]. The prokaryotic *hlyB* gene product is a member of a superfamily of ATP-binding transport proteins [35], located in the inner membrane of *Escherichia coli* and dedicated to the secretion of the toxin (alpha-hemolysin) HlyA in *E. coli* [36]. Therefore, hemolysin may be one of the pathogenic substances of *S. algae *. In addition, the common genes related to type IV pili, which are regulated by external stimuli for directed movement, provided a fresh perspective on pathogenicity [37]. We found that gene *vasF*, which was related to VAS type VI secretion system (T6SS), was specific for *S. algae* strains in group 2 (Fig. 6). T6SS was involved in a variety of cellular processes by secreting effector proteins into the extracellular milieu. Takahiko Ishikawa *et al*. demonstrated that the T6SS of wild-type *V. cholerae* O1 strains was functional and that its expression was controlled by specific environments in a pathogen-adaptive fashion [38], which indicated the *S. algae* strains in group 2 may have great adaptability to the environment.

Different bacteria in the gut flora could compete with other species through contact-dependent mechanisms, such as the T6SS. For example, *Salmonella Typhimurium* could utilize the T6SS to inject effector proteins into target cells to enhance its intestinal colonization ability [39]. *Vibrio cholerae* could use its T6SS to kill the symbiotic bacteria to promote colonization and enhance its pathogenicity, revealing that the absence of symbiotic bacteria may weaken the severity of diseases caused by *V. cholerae,* and *V. cholerae* enhances its pathogenicity through competition with symbiotic bacteria [40]. In this study, most clinical strains did not carry the *vas* gene, which means that clinical strains may not be very competitive for colonization since *S. algae* was just considered as an opportunistic pathogen. Considering *S. algae* is widely distributed in the oceans and has a potential to carry *vas* genes for colonization, it is necessary to strengthen *S. algae* environmental monitoring. Almost all strains contain *katA* gene, the product of which can decompose hydrogen peroxide, protect cells from the toxic effects of hydrogen peroxide, and enhance the bacterial colonization ability in the host [41]. Most of the *S. algae* strains also contained a stress-regulating gene *sodCI*. This gene encodes superoxide dismutase, which can destroy free radicals produced by host cells so as to eradicate bacteria, and enhance the bacterial resistance [42]. In our study, the genomes of 45 strains contained *rmlA* gene, which was related to capsular polysaccharide. It has been demonstrated that the mutations of the *rmlA* gene reduced biofilm-forming capacity of *Stenotrophomonas maltophilia* [43]. Our study showed that the ratio of *rmlA* gene in environmental *S. algae* strains was higher than that in its clinical counterparts, which revealed new insights into the environmental adaptability of *S. algae *. Two strains (strains 08MAS2314 and JFC1) contained urease-related genes *ureB* and *ureG*. Fu *et al*. demonstrated that the products of *ureB* and *ureG* genes are important virulence factors of *Vibrio harveyi* and enhance the pathogenicity to fish [44]. Also, Mehta *et al*. confirmed *ureG* gene could encode urease, which was essential for the colonization of *Helicobacter pylori* in stomach [45]. Most *Brucella spp*. also show strong urease activity. The products encoded by *ureB* and *ureG* are involved in protecting *Brucella spp*. from gastric acid and thus contribute to the pathogenicity [46]. Therefore, these two genes may also play an important role in the pathogenic process of *S. algae *. O-antigen, a virulence factor of Gram-negative bacteria, is the outermost structure of lipopolysaccharide. O-antigen is important for environmental adaptability because it is the main target of bacteriophages and the host immune system. O-antigen can induce a strong immune response in the host [47]. In our study, 19 strains carry the O-antigen synthesis-related genes. At the current stage, we failed to effectively detect the relatedness between virulence-associated genes and the sources of *S. algae* strains, suggesting that there may be minimal difference between clinical and environmental stains in terms of virulence-associated genes. Thus, we inferred that the pathogenicity of *S. algae* is affected by many factors such as complex interaction between the host and the environment, which is in accordance with the report of Janda *et al*. [1].

It is increasingly agreed that *S. algae* is a dominant human pathogen in the genus *Shewanella* when people are exposed to marine niches containing this pathogen through occupational or recreational activities. Documented illnesses linked to *S. algae* included skin and soft tissue infections, bacteremia, and otitis media. Generally, *S. algae* mostly infect people with an impaired immune system [2, 48, 49]. Also, we found no clear distinction in (potential) virulence determinants between environmental and clinical isolates of *S. algae *, which suggests that most *S. algae* strains may only cause opportunistic infections. Accordingly, we could reasonably speculate that the infection of *S. algae* is a preliminary condition rather than a decisive factor for human disease, and the pathogenic process is the result of the synergistic effect of multiple factors.

GIs harbouring a cluster of genes, are defined as probable horizontal origins in bacterial or archaeal genomes [27]. As one of the major drivers of genome evolution, GI can enhance the fitness of bacteria within a niche. There were 167 GIs and 65 phage-related elements detected in our study. Four GIs were associated with cross-species horizontal transfer. The GIs detected in strain 2NE11 were highly homologous to those in *Halomonas meridiana* Eplume2 and *Alcanivorax* N3-2A. Also, two GIs, which were detected in strains RQs-106 and CCU101 respectively, were highly homologous to those in *Aeromonas hydrophila* ONP3.1 and *Marinobacter hydrocarbonoclasticus* VT8 respectively. The sequences of prophage-related elements detected in strain 18064-CSB-B-B were 100 % similar to those in *E. coli* AR435 and *Salmonella* SL131. The GIs carried a suite of virulence genes and mobile elements, and the prophages contained genes encoding transposase and integrase. The horizontal gene transfer across species breaks down the boundaries of kinship and makes gene flow more complex. Therefore, we speculated that the cross-species gene transfer caused by GIs or prophage-related elements contributes to the evolution and independent acquisitions of virulence factors of *S. algae *. In addition, strains 2NE11, VGH117 and RQs-106 harboured GIs that encode several acyltransferases. Acyltransferases are enzymes which transfer acyl groups to specific targets and may be an important factor regulating the production of virulence factors, motility and biofilm formation.

The CRISPR-Cas system is a prokaryotic adaptive immune system which has evolved to get rid of foreign invading genes from viruses. The system is found in many bacteria, showing great complexity and diversity [50]. In our study, 23 strains harboured CRISPR-Cas system with CAS-TypeIE and CAS-TypeIF as the main Cas types. It is worth pointing out that the strain C6G3 has an incomplete CRISPR-Cas system, which means that this strain may have a defect in its natural immune defenses process.

Related literature reported that the aquatic bacterium *Shewanella* was a reservoir for MCR-4 mobile colistin resistance [51], and *S. algae* was the source of plasmid-mediated QnrA determinants [52]. A variety of drug-resistant genetic elements were identified in some *Shewanella* spp. with environmental or clinical origins, highlighting that the genus is probably a vehicle and a reservoir of antibiotic resistance genes [9, 10, 53–55]. These genes showed resistance to different antibiotic classes, including β-lactams, quinolones, aminoglycosides, macrolides and carbapenems. In this study, all 55 *S *. *algae* strains were found to carry antibiotic resistance genes, among which the carriage rate of *bla*_OXA_ gene was 100 %, and that of *qnr* gene followed. It is worth noting that the plasmids of strains 18064-CSB-B-B and CCU101 were proved to carry RTFs, which contribute to the spread of antibiotic-resistance genes. Over the past few years, various investigations have extensively reported drug-resistant (XDR) *Shewanella* species from environmental and human infection samples, like XDR *S. xiamenensis [10]*. Among the 55* S. algae* strains in our study, 67.3 % contained genes associated with resistance to more than one class of antibiotics (strain KC-NA-R1 carried the most number: 14). In addition, clinical strains have a higher frequency of carrying resistance genes of aminoglycosides (21.9%) compared with environmental strains (8.7 %), which may reveal an early warning for clinical surveillance and treatment of *S. algae* infection, since aminoglycosides are more often preferred in the treatment of severe infection caused by aerobic Gram-negative bacteria.

It is worth noting that, the drug-resistant phenotype of the 55 strains in our study did not match the drug-resistant genes carried in their genomes. Several reasons may account for this phenomenon. First, the genomes of most strains were not complete so the drug-resistant genes may be interrupted. Also, although the antimicrobial resistant phenotypes were determined by the corresponding genotypes, there was no absolute consistency between them [56, 57]. Jianhua Yin *et al*. discovered that the *blaA* gene was strongly induced by ampicillin at high (50 µg ml^−1^), but not at low levels (2.5 µg ml^−1^) [58]. These reasons may explain part of the mismatch that the 12 clinical strains in this study had a high frequency of carrying the resistance genes of aminoglycosides but were all sensitive to four tested aminoglycoside drugs.

## Supplementary Data

Supplementary material 1
