# Supplementary material for: Genomic analysis reveals high intra-species diversity of Shewanella algae
Source: Microb Genom. 2022 Feb 10;8(2):000786. doi: 10.1099/mgen.0.000786 (PMC8942018; doi:10.1099/mgen.0.000786)
Supplement: Supplementary material 1 [file mgen-8-0786-s001.pdf]

**Supplementary files**

**Table S1** Detailed information of 55 *Shewanella algae* strains.

| No. | Strain | Region | Source | Year | Accession No. |
|---|---|---|---|---|---|
| 1 | 18064-CSB-B-B | Tanzania | poultry stool | 2018 | GCA_009846595.1 |
| 2 | 20-23R | South Korea | intestine of *upeneus japonicus* | 2015 | GCA_002836995.1 |
| 3 | 2NE11 | Peru | olive production company | 2018 | GCA_014263185.1 |
| 4 | 38A_GOM_205m | - | Deepwater Horizon oil rig explosion | - | GCA_000518445.1 |
| 5 | AC | Taiwan, China | *Haliotis diversicolor* | 2014 | GCA_003024535.1 |
| 6 | ATCC 51192 | France | Red alga | - | GCA_012396675.1 |
| 7 | C6G3 | France | marine sediment | 2007 | GCA_000956365.1 |
| 8 | CECT 5071 | Japan | Jania sp. (Red alga) | 1990 | GCA_009183365.1 |
| 9 | CSB04KR | South Korea | gut of *Apostichopus japonicus* | 2015 | GCA_001858195.1 |
| 10 | Iso12 | USA | estuarine water | 2013 | GCA_012030025.1 |
| 11 | JCM 14758 | Korea | gut microflora of abalone | 2006 | GCA_000614935.1 |
| 12 | JCM 21037 | Japan | Alga (*Jania* sp.) | - | GCA_000615045.1 |
| 13 | JFC1 | Taiwan, China | *Crassostrea gigas* | 2014 | GCA_007860205.1 |
| 14 | JFC2 | Taiwan, China | *Crassostrea gigas* | 2014 | GCA_007844895.1 |
| 15 | JFC3 | Taiwan, China | *Crassostrea gigas* | 2014 | GCA_007636355.1 |
| 16 | KC-Na-R1 | South Korea | Neophocaena phocaenoides | 2017 | GCA_003721455.1 |
| 17 | MN-01 | USA | Salt marsh sediment | 2010 | GCA_002237105.1 |
| 18 | NBRC 103173 | Japan | Alga (*Jania* sp.) | - | GCA_001598875.1 |
| 19 | RQs-106 | Liaoning, China | activated sludge | 2011 | GCA_009730655.1 |
| 20 | SYT1 | Taiwan, China | *Haliotis diversicolor* | 2014 | GCA_007595095.1 |
| 21 | SYT2 | Taiwan, China | *Crassostrea gigas* | 2014 | GCA_007595325.1 |
| 22 | SYT3 | Taiwan, China | Seawater | 2014 | GCA_007595405.1 |
| 23 | SYT4 | Taiwan, China | *Meretrix lusoria* | 2014 | GCA_003024575.1 |
| 24 | ACCC | Taiwan, China | patient, bile | 2014 | GCA_003025175.1 |
| 25 | CCU101 | Taiwan, China | patient, abscess | 2013 | GCA_003427415.1 |
| 26 | CHL | Taiwan, China | patient, bile | 2014 | GCA_007595205.1 |
| 27 | CLS1 | Taiwan, China | patient, wound | 2014 | GCA_007595085.1 |
| 28 | CLS2 | Taiwan, China | patient, blood | 2014 | GCA_007595365.1 |
| 29 | CLS3 | Taiwan, China | patient, blood | 2014 | GCA_007595375.1 |
| 30 | CLS4 | Taiwan, China | patient, blood | 2014 | GCA_007595165.1 |
| 31 | CLS5 | Taiwan, China | patient, bile | 2014 | GCA_007595175.1 |
| 32 | JFL | Taiwan, China | patient, blood | 2014 | GCA_007636455.1 |
| 33 | MARS-14 | France | patient, pneumonia | - | GCA_000947195.1 |
| 34 | melkephyllucas | Taiwan, China | patient, blood | 2014 | GCA_007595215.1 |
| 35 | NCTC 10738 | - | patient, stool | 1970 | GCA_900457125.1 |

| | | | | | |
|---|---|---|---|---|---|
| 36 | RC | Taiwan, China | patient, blood | 2014 | GCA_007636395.1 |
| 37 | Sh392 | Argentina | patient, skin and soft tissue infection | 2006 | GCA_003124085.1 |
| 38 | SYC | Taiwan, China | patient, blood | 2014 | GCA_008119825.1 |
| 39 | TYL | Taiwan, China | patient, blood | 2014 | GCA_007636495.1 |
| 40 | VGH117 | Taiwan, China | patient, tissue infection | 2015 | GCA_014702225.1 |
| 41 | YHL | Taiwan, China | patient, wound | 2014 | GCA_002318995.1 |
| 42 | YTH | Taiwan, China | patient, blood | 2014 | GCA_007636365.1 |
| 43 | YTL | Taiwan, China | patient, blood | 2014 | GCA_007636415.1 |
| 44 | 08MAS2314 | Anhui, China | patient, stool | 2008 | JAGQAT000000000 |
| 45 | LC2016-1 | Shandong, China | patient, pneumonia | 2016 | JAGQAS000000000 |
| 46 | LC2016-2 | Shandong, China | patient, pneumonia | 2016 | JAGQAR000000000 |
| 47 | LC2016-3 | Shandong, China | patient, pneumonia | 2016 | JAGQAQ000000000 |
| 48 | LC2016-4 | Shandong, China | patient, pneumonia | 2016 | JAGQAP000000000 |
| 49 | LC2016-5 | Shandong, China | patient, blood | 2016 | JAGQAO000000000 |
| 50 | LC2016-6 | Shandong, China | patient, blood | 2016 | JAGQAN000000000 |
| 51 | LZ201228 | Shandong, China | patient, stool | 2012 | JAGQAM000000000 |
| 52 | LZ2013652 | Shandong, China | patient | 2013 | JAGQAL000000000 |
| 53 | LZ2015243 | Shandong, China | patient | 2015 | JAGQAK000000000 |
| 54 | LZ2015256 | Shandong, China | patient, diarrhea | 2015 | JAGQAJ000000000 |
| 55 | MAS2736 | Anhui, China | patient, diarrhea | 2007 | JAGQAI000000000 |

Note: The No. 1-23 strains are environmental isolates; No. 24-55 strains are clinical isolates.

**Table S2** Detailed information of 55 *S. algae* genomes.

| No. | Strain | Contigs /Scaffolds | N50 size (bp) | Longest scaffold (bp) | Size (Mb) | G+C (mol%) | CDS | tRNA | Level | Accession No. |
|---|---|---|---|---|---|---|---|---|---|---|
| 1 | 18064-CSB-B-B | 2 | - | 4754321 | 4.90 | 53.0 | 4361 | 106 | Complete | GCA_009846595.1 |
| 2 | 20-23R | 56 | 165375 | 487435 | 4.76 | 53.1 | 4194 | 113 | Draft | GCA_002836995.1 |
| 3 | 2NE11 | 1 | - | 5030813 | 5.03 | 53.0 | 4387 | 109 | Complete | GCA_014263185.1 |
| 4 | 38A_GOM_205m | 57 | 147064 | 404292 | 4.74 | 53.1 | 4183 | 70 | Draft | GCA_000518445.1 |
| 5 | AC | 28 | 289615 | 663370 | 4.75 | 53.1 | 4196 | 86 | Draft | GCA_003024535.1 |
| 6 | ATCC 51192 | 52 | 224036 | 1051072 | 4.98 | 52.5 | 4425 | 94 | Draft | GCA_012396675.1 |
| 7 | C6G3 | 43 | 336799 | 498365 | 4.88 | 53.1 | 5694 | 85 | Draft | GCA_000956365.1 |
| 8 | CECT 5071 | 36 | 446958 | 1050331 | 4.87 | 53.0 | 4307 | 87 | Draft | GCA_009183365.1 |
| 9 | CSB04KR | 64 | 134937 | 301346 | 4.80 | 53.0 | 4269 | 91 | Draft | GCA_001858195.1 |
| 10 | Iso12 | 57 | 415377 | 916664 | 4.97 | 52.9 | 4465 | 92 | Draft | GCA_012030025.1 |
| 11 | JCM 14758 | 125 | 68021 | 233475 | 4.93 | 52.9 | 5416 | 60 | Draft | GCA_000614935.1 |
| 12 | JCM 21037 | 108 | 101380 | 305083 | 4.83 | 53.0 | 5154 | 64 | Draft | GCA_000615045.1 |
| 13 | JFC1 | 91 | 98142 | 269396 | 4.80 | 53.0 | 4294 | 94 | Draft | GCA_007860205.1 |
| 14 | JFC2 | 44 | 185366 | 689388 | 4.84 | 53.0 | 4262 | 89 | Draft | GCA_007844895.1 |
| 15 | JFC3 | 43 | 220614 | 662381 | 4.82 | 53.0 | 4238 | 92 | Draft | GCA_007636355.1 |
| 16 | KC-Na-R1 | 2 | - | 5036300 | 5.20 | 52.8 | 4621 | 104 | Complete | GCA_003721455.1 |
| 17 | MN-01 | 102 | 80731 | 284248 | 4.77 | 53.0 | 4189 | 70 | Draft | GCA_002237105.1 |
| 18 | NBRC 103173 | 143 | 65053 | 155184 | 4.82 | 53.1 | 4277 | 58 | Draft | GCA_001598875.1 |
| 19 | RQs-106 | 1 | - | 4990025 | 4.99 | 53.0 | 4353 | 105 | Complete | GCA_009730655.1 |
| 20 | SYT1 | 103 | 108667 | 309963 | 4.81 | 53.1 | 4252 | 89 | Draft | GCA_007595095.1 |
| 21 | SYT2 | 37 | 294530 | 685673 | 4.78 | 53.1 | 4240 | 83 | Draft | GCA_007595325.1 |
| 22 | SYT3 | 80 | 136155 | 259860 | 4.82 | 53.0 | 4279 | 67 | Draft | GCA_007595405.1 |
| 23 | SYT4 | 37 | 201209 | 884367 | 4.84 | 53.1 | 4266 | 89 | Draft | GCA_003024575.1 |

| | | | | | | | | | | |
|---|---|---|---|---|---|---|---|---|---|---|
| 24 | ACCC | 74 | 112688 | 589495 | 4.74 | 53.1 | 4247 | 90 | Draft | GCA_003025175.1 |
| 25 | CCU101 | 2 | - | 4786791 | 4.92 | 53.1 | 4653 | 114 | Complete | GCA_003427415.1 |
| 26 | CHL | 55 | 162092 | 429624 | 4.89 | 53.0 | 4347 | 88 | Draft | GCA_007595205.1 |
| 27 | CLS1 | 159 | 70370 | 217863 | 4.89 | 53.0 | 4431 | 90 | Draft | GCA_007595085.1 |
| 28 | CLS2 | 115 | 78097 | 281057 | 4.88 | 52.9 | 4299 | 89 | Draft | GCA_007595365.1 |
| 29 | CLS3 | 128 | 56019 | 172630 | 4.60 | 53.1 | 4105 | 69 | Draft | GCA_007595375.1 |
| 30 | CLS4 | 35 | 199034 | 518903 | 4.87 | 53.0 | 4384 | 89 | Draft | GCA_007595165.1 |
| 31 | CLS5 | 43 | 184153 | 381410 | 4.82 | 53.0 | 4298 | 92 | Draft | GCA_007595175.1 |
| 32 | JFL | 41 | 230173 | 996788 | 4.80 | 53.1 | 4250 | 90 | Draft | GCA_007636455.1 |
| 33 | MARS-14 | 11 | 551728 | 1135015 | 5.01 | 52.9 | 4382 | 106 | Draft | GCA_000947195.1 |
| 34 | melkephyllucas | 51 | 209797 | 577344 | 4.81 | 53.1 | 4269 | 83 | Draft | GCA_007595215.1 |
| 35 | NCTC 10738 | 3 | 1829301 | 3159547 | 5.00 | 52.8 | 4424 | 108 | Draft | GCA_900457125.1 |
| 36 | RC | 54 | 164679 | 662666 | 4.81 | 53.0 | 4232 | 88 | Draft | GCA_007636395.1 |
| 37 | Sh392 | 153 | 91550 | 367520 | 4.80 | 52.9 | 4255 | 105 | Draft | GCA_003124085.1 |
| 38 | SYC | 49 | 159600 | 386020 | 4.77 | 52.9 | 4239 | 82 | Draft | GCA_008119825.1 |
| 39 | TYL | 100 | 96168 | 234074 | 4.82 | 53.0 | 4252 | 89 | Draft | GCA_007636495.1 |
| 40 | VGH117 | 1 | - | 4796801 | 4.80 | 53.1 | 4182 | 107 | Complete | GCA_014702225.1 |
| 41 | YHL | 27 | 271847 | 976090 | 4.85 | 53.0 | 4261 | 86 | Draft | GCA_002318995.1 |
| 42 | YTH | 52 | 137161 | 662669 | 4.81 | 53.0 | 4237 | 89 | Draft | GCA_007636365.1 |
| 43 | YTL | 60 | 133967 | 438632 | 4.79 | 52.9 | 4268 | 82 | Draft | GCA_007636415.1 |
| 44 | 08MAS2314* | 57 | 188182 | 438576 | 4.79 | 53.1 | 4219 | 97 | Draft | JAGQAT000000000 |
| 45 | LC2016-1* | 120 | 87342 | 282758 | 4.68 | 53.1 | 4170 | 112 | Draft | JAGQAS000000000 |
| 46 | LC2016-2* | 156 | 66841 | 203209 | 4.66 | 53.1 | 4162 | 50 | Draft | JAGQAR000000000 |
| 47 | LC2016-3* | 159 | 67674 | 166246 | 4.66 | 53.1 | 4157 | 50 | Draft | JAGQAQ000000000 |
| 48 | LC2016-4* | 117 | 87235 | 282777 | 4.68 | 53.1 | 4168 | 112 | Draft | JAGQAP000000000 |
| 49 | LC2016-5* | 87 | 118094 | 256215 | 4.86 | 52.9 | 4394 | 108 | Draft | JAGQAO000000000 |

| | | | | | | | | | | |
|---|---|---|---|---|---|---|---|---|---|---|
| 50 | LC2016-6* | 105 | 98511 | 223179 | 4.84 | 52.9 | 4383 | 45 | Draft | JAGQAN000000000 |
| 51 | LZ201228* | 34 | 366387 | 773111 | 4.87 | 53.1 | 4297 | 113 | Draft | JAGQAM000000000 |
| 52 | LZ2013652* | 95 | 100763 | 288096 | 4.71 | 53.1 | 4199 | 56 | Draft | JAGQAL000000000 |
| 53 | LZ2015243* | 89 | 97980 | 419980 | 4.80 | 53.0 | 4285 | 95 | Draft | JAGQAK000000000 |
| 54 | LZ2015256* | 160 | 63613 | 216271 | 4.77 | 53.0 | 4259 | 97 | Draft | JAGQAJ000000000 |
| 55 | MAS2736* | 38 | 103702 | 297130 | 4.81 | 53.0 | 4247 | 93 | Draft | JAGQAI000000000 |

Note: The No. 1-23 strains are environmental isolates; No. 24-55 strains are clinical isolates. The strains genomes obtained in this study were marked with “*”.

**Table S3** Unique prophage-related sequences distributed in *S. algae* strains.

| Strain | Prophage | Size (kb) | Total Protein Num | G+C mol/% | Status | Specific Keywords |
|---|---|---|---|---|---|---|
| 18064-CSB-B-B | 1 | 12.9 | 10 | 54.28 | incomplete | transposase |
| | 2 | 6.5 | 10 | 51.70 | incomplete | transposase |
| KC-Na-R1 | 3 | 21.7 | 25 | 51.23 | incomplete | tail,integrase |
| | 4 | 32.3 | 23 | 50.39 | questionable | portal, protease, tail, plate, integrase |
| | 5 | 40.7 | 40 | 51.65 | intact | integrase, transposase, portal, virion, protease, head, tail, plate |
| | 6 | 35.4 | 44 | 50.00 | intact | tail, capsid, protease, virion, portal, transposase |
| 2NE11 | 7 | 34.6 | 19 | 43.75 | incomplete | tail |
| RQs-106 | 8 | 42 | 46 | 52.47 | intact | lysin, integrase, head, portal, capsid, terminase, tail |
| ATCC 51192 | 9 | 6.6 | 7 | 52.78 | incomplete | protease |
| CECT 5071 | 10 | 6.6 | 7 | 46.25 | incomplete | tail |

| | | | | | | |
|---|---|---|---|---|---|---|
| | 11 | 13.9 | 24 | 48.04 | incomplete | integrase, tail |
| AC | 12 | 9.8 | 9 | 51.79 | incomplete | tail, protease |
| SYT4 | 13 | 30.9 | 15 | 45.72 | incomplete | tail, transposase |
| JFC2 | 14 | 8.8 | 7 | 50.44 | incomplete | unknown |
| SYT1 | 15 | 8.2 | 8 | 48.29 | incomplete | transposase, tail |
| JFC1 | 16 | 19.2 | 8 | 49.66 | incomplete | integrase, transposase |
| Iso12 | 17 | 15.7 | 27 | 47.28 | incomplete | integrase, tail |
| 38A_GOM_205m | 18 | 10 | 12 | 43.42 | incomplete | tail |
| CCU101 | 19 | 9 | 9 | 50.50 | incomplete | transposase |
| | 20 | 8.9 | 10 | 52.25 | incomplete | transposase |
| | 21 | 8.8 | 9 | 39.19 | incomplete | transposase |
| YHL | 22 | 27.5 | 16 | 48.02 | incomplete | tail |
| JFL | 23 | 20 | 23 | 57.29 | intact | tail, lysin, head, terminase, capsid, portal |
| CLS5 | 24 | 22.9 | 27 | 56.68 | incomplete | lysin |
| | 25 | 13.7 | 18 | 48.43 | incomplete | integrase, tail, transposase |
| ACCC | 26 | 36.7 | 31 | 51.72 | questionable | integrase, plate, tail, head, portal |
| | 27 | 10.3 | 21 | 50.22 | incomplete | capsid |
| NCTC 10738 | 28 | 15.9 | 14 | 45.58 | incomplete | tail, transposase, integrase |
| | 29 | 5.8 | 10 | 47.41 | incomplete | portal, transposase |
| | 30 | 7.2 | 9 | 48.68 | incomplete | transposase |
| | 31 | 4.4 | 8 | 52.52 | incomplete | transposase, tail |
| melkephyllucas | 32 | 41.5 | 45 | 51.80 | intact | integrase, transposase, portal, virion, protease, head, tail, plate |
| TYL | 33 | 6.7 | 7 | 37.92 | incomplete | unknown |
| CLS2 | 34 | 8.4 | 8 | 47.76 | incomplete | unknown |

| | | | | | | |
|---|---|---|---|---|---|---|
| | 35 | 31.5 | 46 | 53.67 | incomplete | lysin |
| CLS4 | 36 | 27.1 | 26 | 51.66 | incomplete | integrase, capsid, tail |
| | 37 | 14.8 | 18 | 51.33 | incomplete | portal |
| | 38 | 30.8 | 6 | 49.63 | incomplete | integrase, transposase |
| | 39 | 7.1 | 7 | 49.56 | incomplete | transposase |
| YTL | 40 | 9.8 | 9 | 51.81 | incomplete | protease, tail |

**Table S4** Shared prophage-related sequences distributed in *S. algae* strains.

| Prophage | Strain | Size (kb) | Total Protein Num | G+C mol/% | Status | Specific Keywords |
|---|---|---|---|---|---|---|
| 1 | JCM 21037; ATCC 51192 | 13.9 | 24 | 48.04 | incomplete | integrase, tail |
| 2 | SYT1; NCTC 10738 | 8.8 | 7 | 50.42 | incomplete | unknown |
| 3 | YTH; YHL; VGH117; TYL; SYT4; SYT2; SYC; melkephyllucas; JFL; JFC2; JFC3; Iso12; CLS4; CHL; ATCC 51192; ACCC; 18064-CSB-B-B | 9.8 | 9 | 51.86 | incomplete | protease, tail |
| 4 | ATCC 51192; CECT 5071 | 6.6 | 7 | 52.78 | incomplete | protease |
| 5 | CLS1; JFC1 | 6.6 | 8 | 46.00 | incomplete | tail |

**Table S5** Predicted plasmid replicons distributed in *S. algae* strains.

| Strain | Source | Plasmid replicon | Identity (%) | Coverage (bp) | Accession No. | Plasmid Size (bp)/ Scaffold | Antimicrobial resistance genes | Functions of Productions (encoded by the Virulence-associated genes) |
|---|---|---|---|---|---|---|---|---|
| 18064-CSB-B-B# | Tanzania, poultry stool | IncC_1 | 100 | 417 | JN157804 | 149,553 | *arm*A, *bla*$_{CTX\text{-}M\text{-}15}$, *sul1*, *dfrA12* | conjugative transfer protein, Phage recombination protein Bet, Mobile element protein, Antitoxin HigA, Toxin HigB |
| CCU101# | Taiwan, China, patient | IncC_1 | 100 | 417 | JN157804 | 132,546 | *aph(3'')-Ib*, *aph(6)-Id*, *floR*, *sul2* | Integrase, Antitoxin HigA, Toxin HigB, Mobile element protein, Type IV secretory pathway, Chromosome (plasmid) partitioning protein ParB, plasmid Conjugative transfer protein, |
| CLS1 | Taiwan, China, patient | IncC_1 | 100 | 417 | JN157804 | -/NZ_LTBI01000028.1 | - | - |
| | | Col440I_1 | 94.737 | 114 | CP023920 | -/NZ_LTBI01000158.1 | - | - |
| JCM 14758 | Korea, gut of abalone | IncC_1 | 97.362 | 417 | JN157804 | -/NZ_BALL01000004.1 | - | - |
| LC2016_5 | Shandong, China, patient | IncU_1 | 100 | 565 | DQ401103 | -/scaffold19 | - | - |
| LC2016_6 | Shandong, China, patient | IncU_1 | 100 | 565 | DQ401103 | -/NODE_85_length_2226_cov_579.241824 | - | - |

Note: # represents complete genome, that is, the plasmid size could be measured.

**Table S6** General information of CRISPR/Cas systems detected in the 23 strains.

| Strain | CRISPR Id | Cas Type | Element | Start | End | Spacer /Gene | Repeat consensus /cas genes |
|---|---|---|---|---|---|---|---|
| 08MAS2314 | scaffold9 | CAS-TypeIF | CRISPR | 40019 | 42625 | 43 | GTTCACTGCCGCCCAGGCAGCTTAGAAA |
| | | | Cas cluster | 31288 | 39868 | 6 | Cas1_0_IF, Cas3-Cas2_0_IF, Csy1_0_IF, Csy2_0_IF, Csy3_0_IF, Cas6_0_IF |
| NCTC10738 | NZ_UGYO01000001.1 | CAS-TypeIE | CRISPR | 2290760 | 2294878 | 67 | GTGTTCCCCGCACTCACGGGGATAAACCG |
| | | | Cas cluster | 2281558 | 2290661 | 8 | Cas3_0_I, Cse1_0_IE, Cse2_0_IE, Cas7_0_IE, Cas5_0_IE, Cas6_0_IE, Cas1_0_IE, Cas2_0_IE |
| LZ2013652 | NODE_8_length_140844_cov192.791631.1 | CAS-TypeIF | CRISPR | 97372 | 104720 | 122 | TTTCTAAGCTGCCTGGGCGGCAGTGAAC |
| | | | Cas cluster | 97372 | 104720 | 6 | Cas6_0_IF, Csy3_0_IF, Csy2_0_IF, Csy1_0_IF, Cas3-Cas2_0_IF, Cas1_0_IF |
| 2NE11 | complete genome | CAS-TypeIF | CRISPR | 4481737 | 4484703 | 49 | TTTCTAAGCTGCCTGGGCGGCAGTGAAC |
| | | | Cas cluster | 4484854 | 4493434 | 6 | Cas6_0_IF, Csy3_0_IF, Csy2_0_IF, Csy1_0_IF, Cas3-Cas2_0_IF, Cas1_0_IF |
| melkephyllucas | scaffold4 | CAS-TypeIF | CRISPR | 279802 | 282529 | 45 | TTTCTAAGCTGCCTGGGCGGCAGTGAAC |
| | | | Cas cluster | 282680 | 291260 | 6 | Cas6_0_IF, Csy3_0_IF, Csy2_0_IF, Csy1_0_IF, Cas3-Cas2_0_IF, Cas1_0_IF |
| 20-23R | scaffold6 | CAS-TypeIF | CRISPR | 41475 | 45946 | 74 | GTTCACTGCCGCCCAGGCAGCTTAGAAA |
| | | | Cas cluster | 32750 | 41324 | 6 | Cas1_0_IF, Cas3-Cas2_0_IF, Csy1_0_IF, Csy2_0_IF, Csy3_0_IF, Cas6_0_IF |
| AC | scaffold2 | CAS-TypeIF | CRISPR | 621411 | 625219 | 63 | TTTCTAAGCTGCCTGGGCGGCAGTGAAC |
| | | | Cas cluster | 625370 | 633585 | 6 | Cas6_0_IF, Csy3_0_IF, Csy2_0_IF, Csy1_0_IF, Cas3-Cas2_0_IF, Cas1_0_IF |
| CCU101 | complete genome | | CRISPR | 1099894 | 1102981 | 51 | TTTCTAAGCTGCCTGGGCGGCAGTGAAC |

| | | | | | | | |
|---|---|---|---|---|---|---|---|
| | | CAS-TypeIF | Cas cluster | 1103132 | 1111347 | 6 | Cas6_0_IF, Csy3_0_IF, Csy2_0_IF, Csy1_0_IF, Cas3-Cas2_0_IF, Cas1_0_IF |
| KC-Na-R1 | complete genome | | CRISPR | 4748730 | 4753439 | 78 | TTTCTAAGCTGCCTGGGCGGCAGTGAAC |
| | | CAS-TypeIF | Cas cluster | 4753590 | 4762164 | 6 | Cas6_0_IF, Csy3_0_IF, Csy2_0_IF, Csy1_0_IF, Cas3-Cas2_0_IF, Cas1_0_IF |
| ACCC | scaffold8 | | CRISPR | 44837 | 47685 | 47 | GTTCACTGCCGCCCAGGCAGCTTAGAAA |
| | | CAS-TypeIF | Cas cluster | 35659 | 43874 | 6 | Cas1_0_IF, Cas3-Cas2_0_IF, Csy1_0_IF, Csy2_0_IF, Csy3_0_IF, Cas6_0_IF |
| C6G3 | Contig_102 | - | CRISPR | 33 | 857 | 13 | GTTCACTGCCGCCCAGGCAGCTTAGAAA |
| | Contig_45 | - | CRISPR | 28 | 414 | 6 | GTTCACTGCCGCCCAGGCAGCTTAGAAA |
| | Contig_86 | - | CRISPR | 57 | 1234 | 19 | GTTCACTGCCGCCCAGGCAGCTTAGAAA |
| MARS-14 | NZ_LN810019.1 | CAS-TypeIF | CRISPR | 3995461 | 3999627 | 69 | TTTCTAAGCTGCCTGGGCGGCAGTGAAC |
| | | | Cas cluster | 3999778 | 4008720 | 6 | Cas6_0_IF, Csy3_0_IF, Csy2_0_IF, Csy1_0_IF, Cas3-Cas2_0_IF, Cas1_0_IF |
| RQs-106 | complete genome | CAS-TypeIF | CRISPR | 548223 | 553614 | 89 | GTTCACTGCCGCCCAGGCAGCTTAGAAA |
| | | | Cas cluster | 539130 | 548072 | 6 | Cas1_0_IF, Cas3-Cas2_0_IF, Csy1_0_IF, Csy2_0_IF, Csy3_0_IF, Cas6_0_IF |
| CHL | scaffold1 | CAS-TypeIE | CRISPR | 386409 | 393152 | 110 | CGGTTTATCCCCGTGGGTGCGGGGAACAC |
| | | | Cas cluster | 393251 | 402028 | 8 | Cas2_0_IE, Cas1_0_IE, Cas6_0_IE, Cas5_0_IE, Cas7_0_IE, Cse2_0_IE, Cse1_0_IE, Cas3_0_I |
| JFC2 | scaffold3 | CAS-TypeIE | CRISPR | 39497 | 41234 | 28 | GAGTTCCCCGCACCCACGGGGATAAACCG |
| | | | Cas cluster | 30243 | 39398 | 8 | Cas3_0_I, Cse1_0_IE, Cse2_0_IE, Cas7_0_IE, Cas5_0_IE, Cas6_0_IE, Cas1_0_IE, Cas2_0_IE |
| JFC3 | scaffold5 | CAS-TypeIF | CRISPR | 216103 | 217990 | 31 | TTTCTAAGCTGCCTGGGCGGCAGTGAAC |
| | | | Cas cluster | 218141 | 226721 | 6 | Cas6_0_IF, Csy3_0_IF, Csy2_0_IF, Csy1_0_IF, Cas3-Cas2_0_IF, Cas1_0_IF |

| | | | | | | | |
|---|---|---|---|---|---|---|---|
| JFL | scaffold6 | CAS-TypeIE | CRISPR | 44614 | 48671 | 66 | GTGTTCCCCGCACCCACGGGGATAAACCG |
| | | | Cas cluster | 35738 | 44515 | 8 | Cas3_0_I, Cse1_0_IE, Cse2_0_IE, Cas7_0_IE, Cas5_0_IE, Cas6_0_IE, Cas1_0_IE, Cas2_0_IE |
| RC | scaffold11 | CAS-TypeIF | CRISPR | 38826 | 40473 | 27 | GTTCACTGCCGCCCAGGCAGCTTAGAAA |
| | | | Cas cluster | 30095 | 38675 | 6 | Cas1_0_IF, Cas3-Cas2_0_IF, Csy1_0_IF, Csy2_0_IF, Csy3_0_IF, Cas6_0_IF |
| SYT2 | scaffold3 | CAS-TypeIE | CRISPR | 372934 | 376868 | 64 | CGGTTTATCCCCGTGGGTGCGGGGAACTC |
| | | | Cas cluster | 376967 | 385744 | 8 | Cas2_0_IE, Cas1_0_IE, Cas6_0_IE, Cas5_0_IE, Cas7_0_IE, Cse2_0_IE, Cse1_0_IE, Cas3_0_I |
| SYT3 | NZ_LVDW01000075.1 | CAS-TypeIF | CRISPR | 38547 | 40014 | 24 | GTTCACTGCCGCCCAGGCAGCTTAGAAA |
| | | | Cas cluster | 29815 | 38396 | 5 | Cas1_0_IF, Cas3-Cas2_0_IF, Cas6_0_IF, Csy2_0_IF, Csy3_0_IF |
| YHL | scaffold1 | CAS-TypeIF | CRISPR | 934046 | 936653 | 43 | TTTCTAAGCTGCCTGGGCGGCAGTGAAC |
| | | | Cas cluster | 936804 | 945384 | 6 | Cas6_0_IF, Csy3_0_IF, Csy2_0_IF, Csy1_0_IF, Cas3-Cas2_0_IF, Cas1_0_IF |
| YTH | scaffold5 | CAS-TypeIF | CRISPR | 38878 | 40525 | 27 | GTTCACTGCCGCCCAGGCAGCTTAGAAA |
| | | | Cas cluster | 30147 | 38727 | 6 | Cas1_0_IF, Cas3-Cas2_0_IF, Csy1_0_IF, Csy2_0_IF, Csy3_0_IF, Cas6_0_IF |
| 38A_GOM_205m | scaffold10 | CAS-TypeIF | CRISPR | 113408 | 115416 | 33 | TTTCTAAGCTGCCTGGGCGGCAGTGAAC |
| | | | Cas cluster | 117944 | 126159 | 6 | Cas6_0_IF, Csy3_0_IF, Csy2_0_IF, Csy1_0_IF, Cas3-Cas2_0_IF, Cas1_0_IF |

**Table S7** General information of genomic islands related to cross-species transmission.

| No. | Strain | Source | Size (kb) | Key component | Highest coverage (%) | Highest identity (%) | Possible origin (Genbank accession No.) |
|---|---|---|---|---|---|---|---|
| 1 | 2NE11 | 2018/Peru | 12.3 | Conjugative transfer protein TrbB; CopG domain-containing protein; Coupling protein VirD4; ATPase required for T-DNA transfer; Transcriptional regulator; LysR family | 99 | 84.47 | *Halomonas meridiana* Eplume2 (AP022869.1) |
| 2 | 2NE11 | 2018/Peru | 4.0 | Integrase; Transcriptional regulator; LysR family | 100 | 95.90 | *Alcanivorax sp.* N3-2A (CP022307.1) |
| 3 | RQs-106 | 2011/China | 7.1 | Glycosyltransferase; Maltose O-acetyltransferase | 94 | 93.35 | *Aeromonas hydrophila* OnP3.1 (CP050851.1) |
| 4 | CCU101 | 2013/China | 4.4 | AlpA family phage regulatory protein; replication protein C; stabilization protein; P-type conjugative transfer protein TrbJ | 92 | 97.15 | *Marinobacter hydrocarbonoclasticus* VT8 (CP000514.1) |

**Table S8 Mobile genetic elements (MGEs) in the upstream and downstream 10k bp sequences of predicted drug resistance genes**

| Strain | Drug resistance genes | | | | MGEs | | |
|---|---|---|---|---|---|---|---|
| | Gene | identity | Contig | Position in contig | start | stop | function |
| LC2016-1 | *bla*$_{OXA-55}$ | 99.31 | scaffold30 | 275397..276266 | 282757 | 282101 | Transposase InsG for insertion sequence element IS4 |
| LC2016-1 | *floR* | 98.19 | scaffold56 | 63957..65170 | 54110 | 54307 | Conjugative transfer protein TrbL |
| | | | | | 57290 | 58591 | Il-IS_2, transposase |
| | | | | | 65693 | 66097 | Mobile element protein |
| LC2016-2 | *floR* | 98.19 | NODE_96 | 383..1596 | 3723 | 2728 | Mobile element protein |
| | | | | | 6188 | 5046 | Mobile element protein |
| MAS2736 | *bla*$_{OXA-55}$ | 99.66 | Scaffold2 | 424043..424912 | 417640 | 418011 | Transposase, TnpA |
| 2NE11 | *bla*$_{OXA-SHE}$ | 99.31 | NZ_CP055159.1 | 4102402..4103271 | 4092041 | 4090842 | Integrase |
| | | | | | 4096256 | 4096567 | Transposase, Avin_45320 family |
| 18064-CSB-B-B | *armA* | 100.00 | NZ_CP047421.1 | 69108..69881 | 61555 | 58583 | TnpA transposase |
| | | | | | 62115 | 61558 | Mobile element protein |
| | | | | | 63434 | 62421 | Integron integrase IntI1 |
| | | | | | 67946 | 68782 | Mobile element protein |
| | | | | | 70942 | 71646 | Mobile element protein |
| | | | | | 71732 | 73906 | Mobile element protein |
| | | | | | 74614 | 75618 | Mobile element protein |
| 18064-CSB-B-B | *bla*$_{CTX-M-15}$ | 100.00 | NZ_CP047421.1 | 125656..126531 | 120649 | 121137 | Phage protein |
| | | | | | 128049 | 126787 | Mobile element protein |
| 18064-CSB-B-B | *qnrA3* | 99.70 | NZ_CP047422.1 | 2416195..2416851 | 2408827 | 2407790 | Mobile element protein |
| 18064-CSB-B-B | *sul1* | 100.00 | NZ_CP047421.1 | 64924..65763 | 67946 | 68782 | Mobile element protein |
| | | | | | 70942 | 71646 | Mobile element protein |

| | | | | | | | |
|---|---|---|---|---|---|---|---|
| | | | | | 71732 | 73906 | Mobile element protein |
| | | | | | 61555 | 58583 | TnpA transposase |
| | | | | | 62115 | 61558 | Mobile element protein |
| | | | | | 63434 | 62421 | Integron integrase IntI1 |
| 18064-CSB-B-B | *dfrA12* | 100.00 | NZ_CP047421.1 | 63579..64076 | 2408827 | 2407790 | Mobile element protein |
| | | | | | 67946 | 68782 | Mobile element protein |
| | | | | | 70942 | 71646 | Mobile element protein |
| | | | | | 71732 | 73906 | Mobile element protein |
| | | | | | 61555 | 58583 | TnpA transposase |
| | | | | | 62115 | 61558 | Mobile element protein |
| | | | | | 63434 | 62421 | Integron integrase IntI1 |
| ATCC-51192 | *vga(A)* | 98.47 | NZ_JAAXPX010000033.1 | 696..2264 | 144 | 1 | Mobilization protein |
| CCU101 | *aph(3'')-Ib* | 100.00 | NZ_CP018457.1 | 77900..78703 | 70090 | 71583 | Mobile element protein |
| | | | | | 68921 | 69658 | plasmid replication and transfer functions |
| | | | | | 67585 | 68289 | Mobile element protein |
| CCU101 | *aph(6)-Id* | 100.00 | NZ_CP018457.1 | 77064..77900 | 91149 | 90118 | Mobile element protein |
| CCU101 | $bla_{\text{OXA-SHE}}$ | 98.74 | NZ_CP018456.1 | 734947..735816 | 751121 | 751849 | Transposase, Avin_45320 family |
| | | | | | 751885 | 752457 | Transposase, Avin_45320 family |
| CCU101 | *floR* | 98.19 | NZ_CP018457.1 | 72715..73928 | 70090 | 71583 | Mobile element protein |
| | | | | | 68921 | 69658 | plasmid replication and transfer functions |
| | | | | | 67585 | 68289 | Mobile element protein |
| | | | | | 67034 | 66330 | Mobile element protein |
| | | | | | 65127 | 65615 | Phage protein |

| | | | | | | | |
|---|---|---|---|---|---|---|---|
| CCU101 | *qnrA7* | 100.00 | NZ_CP018456.1 | 879143..879799 | 857679 | 856519 | 4-hydroxybutyrate coenzyme A transferase |
| CHL | *bla*$_{\text{OXA-SHE}}$ | 98.85 | NZ_LVDF01000001.1 | 10087..10956 | 3 | 383 | Transposase InsG for insertion sequence element IS4 |
| CHL | *floR* | 98.35 | NZ_LVDF01000025.1 | 1257..2470 | 811 | 272 | Mobile element protein |
| | | | | | 5095 | 3602 | Mobile element protein |
| | | | | | 6907 | 7215 | Transposase InsN for insertion sequence element IS911 |
| | | | | | 7212 | 8084 | Transposase InsO for insertion sequence element IS911 |
| CLS1 | *aadA2* | 100.00 | NZ_LTBI01000080.1 | 8123..8914 | 3330 | 4844 | Transposase |
| | | | | | 4831 | 5616 | Mobile element protein |
| | | | | | 9964 | 10977 | Integron integrase IntI1 |
| | | | | | 11283 | 11840 | Mobile element protein |
| | | | | | 11843 | 14815 | TnpA transposase |
| | | | | | 14894 | 15898 | Mobile element protein |
| CLS1 | *aph(3'')-Ib* | 100.00 | NZ_LTBI01000025.1 | 11093..11896 | 4792 | 5049 | Mobile element protein |
| | | | | | 8079 | 5074 | Mobile element protein |
| | | | | | 8242 | 8799 | Mobile element protein |
| | | | | | 3 | 278 | Mobile element protein |
| CLS1 | *aph(6)-Id* | 100.00 | NZ_LTBI01000025.1 | 10257..11093 | 4792 | 5049 | Mobile element protein |
| | | | | | 8079 | 5074 | Mobile element protein |
| | | | | | 8242 | 8799 | Mobile element protein |
| | | | | | 3 | 278 | Mobile element protein |
| CLS1 | *bla*$_{\text{CMY-2}}$ | 100.00 | NZ_LTBI01000025.1 | 41113..42258 | 36955 | 38271 | IncF plasmid conjugative transfer |

| | | | | | | | |
|---|---|---|---|---|---|---|---|
| | | | | | | | pilus assembly protein TraB |
| | | | | | 38268 | 38846 | IncF plasmid conjugative transfer pilus assembly protein TraV |
| | | | | | 38859 | 39242 | Conjugative transfer protein TraA |
| | | | | | 39527 | 40789 | Mobile element protein |
| | | | | | 50266 | 52713 | IncF plasmid conjugative transfer pilus assembly protein TraC |
| | | | | | 52728 | 53045 | Conjugative transfer protein 345 |
| CLS1 | *bla*$_{TEM-2}$ | 100.00 | NZ_LTBI01000025.1 | 8982..9842 | 4792 | 5049 | Mobile element protein |
| | | | | | 8079 | 5074 | Mobile element protein |
| | | | | | 8242 | 8799 | Mobile element protein |
| | | | | | 3 | 278 | Mobile element protein |
| CLS1 | *erm(42)* | 99.89 | NZ_LTBI01000025.1 | 930..1841 | 3 | 278 | Mobile element protein |
| | | | | | 4792 | 5049 | Mobile element protein |
| | | | | | 8079 | 5074 | Mobile element protein |
| | | | | | 8242 | 8799 | Mobile element protein |
| CLS1 | *catA1* | 99.70 | NZ_LTBI01000134.1 | 2029..2688 | 213 | 1 | Mobile element protein |
| CLS1 | *sul1* | 100.00 | NZ_LTBI01000080.1 | 6779..7618 | 1 | 252 | Mobile element protein |
| | | | | | 300 | 740 | TniA putative transposase |
| | | | | | 3198 | 1654 | Putative transposase InsK for insertion sequence element IS150 |
| | | | | | 3330 | 4844 | Transposase |
| | | | | | 4831 | 5616 | Mobile element protein |
| | | | | | 9964 | 10977 | Integron integrase IntI1 |
| | | | | | 11283 | 11840 | Mobile element protein |

| | | | | | | | |
|---|---|---|---|---|---|---|---|
| | | | | | 11843 | 14815 | TnpA transposase |
| | | | | | 14894 | 15898 | Mobile element protein |
| CLS1 | *sul2* | 100.00 | NZ_LTBI01000025.1 | 11957..12772 | 4792 | 5049 | Mobile element protein |
| | | | | | 8079 | 5074 | Mobile element protein |
| | | | | | 8242 | 8799 | Mobile element protein |
| JFC3 | *bla*$_{\text{OXA-SHE}}$ | 99.31 | NZ_LVCX01000004.1 | 157088..157957 | 163867 | 165204 | Transposase, Avin_45320 family |
| KC-Na-R1 | *aadA1* | 99.87 | NZ_CP033574.1 | 99743..100534 | 105482 | 106495 | Integron integrase IntI1 |
| | | | | | 106792 | 107349 | Mobile element protein |
| | | | | | 107352 | 110324 | TnpA transposase |
| KC-Na-R1 | *aadA16* | 99.65 | NZ_CP033574.1 | 68856..69701 | 67444 | 66431 | Integron integrase IntI1 |
| | | | | | 66070 | 65510 | Mobile element protein |
| | | | | | 65506 | 62540 | Transposase |
| KC-Na-R1 | *ant(2'')-Ia* | 100.00 | NZ_CP033574.1 | 103733..104266 | 105482 | 106495 | Integron integrase IntI1 |
| | | | | | 106792 | 107349 | Mobile element protein |
| | | | | | 107352 | 110324 | TnpA transposase |
| KC-Na-R1 | *bla*$_{\text{OXA-10}}$ | 100.00 | NZ_CP033574.1 | 100551..101351 | 105482 | 106495 | Integron integrase IntI1 |
| | | | | | 106792 | 107349 | Mobile element protein |
| | | | | | 107352 | 110324 | TnpA transposase |
| KC-Na-R1 | *bla*$_{\text{OXA-SHE}}$ | 99.20 | NZ_CP033575.1 | 4354224..4355093 | 4348314 | 4346977 | Transposase, Avin_45320 family |
| KC-Na-R1 | *bla*$_{\text{VEB-1}}$ | 100.00 | NZ_CP033574.1 | 104411..105310 | 105482 | 106495 | Integron integrase IntI1 |
| | | | | | 106792 | 107349 | Mobile element protein |
| | | | | | 107352 | 110324 | TnpA transposase |
| KC-Na-R1 | *cmlA1* | 99.68 | NZ_CP033574.1 | 101616..102875 | 105482 | 106495 | Integron integrase IntI1 |
| | | | | | 106792 | 107349 | Mobile element protein |
| | | | | | 107352 | 110324 | TnpA transposase |

| | | | | | | | |
|---|---|---|---|---|---|---|---|
| KC-Na-R1 | *ARR-2* | 100.00 | NZ_CP033574.1 | 103196..103648 | 105482 | 106495 | Integron integrase IntI1 |
| | | | | | 106792 | 107349 | Mobile element protein |
| | | | | | 107352 | 110324 | TnpA transposase |
| KC-Na-R1 | *ARR-3* | 100.00 | NZ_CP033574.1 | 67617..68069 | 65506 | 62540 | Transposase |
| | | | | | 66070 | 65510 | Mobile element protein |
| | | | | | 58776 | 57052 | Transposase |
| | | | | | 72587 | 71802 | Mobile element protein |
| | | | | | 74088 | 72574 | Transposase |
| KC-Na-R1 | *sul1* | 100.00 | NZ_CP033574.1 | 96561..97400 | 105482 | 106495 | Integron integrase IntI1 |
| | | | | | 106792 | 107349 | Mobile element protein |
| | | | | | 107352 | 110324 | TnpA transposase |
| KC-Na-R1 | *sul1* | 100.00 | NZ_CP033574.1 | 70159..70998 | 65506 | 62540 | Transposase |
| | | | | | 66070 | 65510 | Mobile element protein |
| | | | | | 67444 | 66431 | Integron integrase IntI1 |
| | | | | | 72587 | 71802 | Mobile element protein |
| | | | | | 74088 | 72574 | Transposase |
| KC-Na-R1 | *tet(A)* | 100.00 | NZ_CP033574.1 | 60253..61452 | 58776 | 57052 | Transposase |
| | | | | | 65506 | 62540 | Transposase |
| | | | | | 66070 | 65510 | Mobile element protein |
| | | | | | 67444 | 66431 | Integron integrase IntI1 |
| KC-Na-R1 | *dfrA27* | 100.00 | NZ_CP033574.1 | 68202..68675 | 67444 | 66431 | Integron integrase IntI1 |
| | | | | | 65506 | 62540 | Transposase |
| | | | | | 66070 | 65510 | Mobile element protein |
| | | | | | 58776 | 57052 | Transposase |
| | | | | | 72587 | 71802 | Mobile element protein |

| | | | | | | | |
|---|---|---|---|---|---|---|---|
| | | | | | 74088 | 72574 | Transposase |
| MARS-14 | *bla*$_{OXA-SHE}$ | 99.08 | NZ_LN810019.1 | 3586015..3586146 | 3579039 | 3580376 | Transposase, Avin_45320 family |
| RC | *bla*$_{OXA-SHE}$ | 99.31 | NZ_LVCZ01000004.1 | 99273..100142 | 106052 | 107389 | Transposase, Avin_45320 family |
| RC | *qnrA3* | 99.54 | NZ_LVCZ01000022.1 | 9367..10023 | 11 | 319 | Mobile element protein |
| RQs-106 | *qnrA4* | 99.39 | NZ_CP046378.1 | 784233..784889 | 770389 | 769373 | Mobile element protein |
| SYC | *bla*$_{OXA-55}$ | 98.62 | NZ_LVDC01000015.1 | 151200..152069 | 152527 | 152177 | Transposase, Avin_45320 family |
| SYT1 | *bla*$_{OXA-SHE}$ | 99.08 | NZ_LUCP01000021.1 | 6713..7582 | 796 | 2 | Transposase, Avin_45320 family |
| SYT4 | *qnrA7* | 99.39 | NZ_LVDK01000020.1 | 35136..35792 | 46730 | 47086 | Mobile element protein |
| YTH | *bla*$_{OXA-SHE}$ | 99.31 | NZ_LVDA01000016.1 | 6707..7576 | 797 | 3 | Transposase, Avin_45320 family |
| YTH | *qnrA3* | 99.54 | NZ_LVDA01000023.1 | 9352..10008 | 2 | 304 | Mobile element protein |
| YTL | *bla*$_{OXA-55}$ | 98.62 | NZ_LVDB01000012.1 | 151198..152067 | 152402 | 152175 | Transposase, Avin_45320 family |
| 38A-GOM-205m | *qnrA3* | 99.70 | JADP01000003.1 | 305904..306560 | 186 | 1 | Transposase, Avin_45320 family |
| Iso12 | *qnrA3* | 99.54 | JAAUHW010000012.1 | 70119..70775 | 83124 | 82153 | Mobile element protein |
| | | | | | 84696 | 84535 | Transposase, IS3/IS911 family |

**Table S9** The results of antimicrobial resistance genes analysis of 12 *S. algae* strains.

| Strain | AMP | AMS | CFZ | CTX | CAZ | CFX | IMI | AZM | FEP | MEM | AMC | TET | DOX | MIN | CHL | SXT | Sul | GEN | AMI | KAN | STR | NAL | CIP | LEV | GEM | AZI | CT | PB |
|---|---|---|---|---|---|---|---|---|---|---|---|---|---|---|---|---|---|---|---|---|---|---|---|---|---|---|---|---|
| LZ201228 | I | S | R | S | S | R | I | S | S | S | R | S | S | S | S | S | S | S | S | S | S | S | S | S | S | S | R | I |
| LZ2013652 | R | I | R | R | S | S | S | R | S | S | I | S | S | S | S | S | S | S | S | S | S | S | S | S | S | S | S | S |
| LZ2015243 | I | S | R | R | S | I | S | R | S | S | I | S | S | S | S | S | S | S | S | S | S | S | S | S | S | S | R | I |
| LZ2015256 | S | S | R | S | S | S | S | R | S | S | S | S | S | S | S | S | I | S | S | S | S | S | S | S | S | S | S | S |
| 08MAS2314 | R | R | R | R | S | R | S | S | S | S | S | S | S | S | S | S | S | S | S | S | S | S | S | S | S | S | I | S |
| MAS2736 | S | S | R | R | S | I | I | R | S | S | S | S | S | S | S | R | S | S | S | S | S | S | S | S | S | S | S | S |
| LZ2015-1 | S | S | R | S | S | S | S | R | S | S | S | R | S | S | I | R | I | S | S | S | S | R | R | R | R | S | S | S |
| LZ2015-2 | S | S | R | S | S | R | S | S | S | S | S | R | S | S | I | R | R | S | S | S | S | R | R | R | R | S | S | S |
| LZ2015-3 | S | S | R | S | R | R | S | R | S | S | S | R | S | S | I | R | I | S | S | S | S | R | R | R | R | S | S | S |
| LZ2015-4 | S | S | R | S | S | R | S | R | S | S | I | I | S | S | I | R | R | S | S | S | S | R | R | I | R | S | S | S |
| LZ2015-5 | R | I | R | S | S | S | I | S | S | S | R | S | S | S | S | S | S | S | S | S | S | R | S | S | S | R | R | R |
| LZ2015-6 | R | I | R | S | S | S | R | S | S | S | R | S | S | S | R | S | S | S | S | S | S | R | I | S | I | S | I | S |
| *E. coli* ATCC 25922 | S | S | S | S | S | S | S | S | S | S | S | S | S | S | S | S | S | S | S | S | S | S | S | S | S | S | S | S |

Note: AMP: Ampicillin; AMS: Ampicillin/sulbactam; CFZ: Cefazolin; CTX: Cefotaxime; CAZ: Ceftazidime; CFX: Cefoxitin; IMI: Imipenem; AZM: Aztreonam; FEP: Cefepime; MEM: Meropenem; AMC: Amoxicillin/Clavulanate; TET: Tetracycline; DOX: Doxycycline; MIN: Minocycline; CHL: Chloramphenicol; SXT: Trimethoprim sulfamethoxazole; Sul: Sulfisoxazole; GEN: Gentamicin; AMI: Amikacin; KAN: Kanamycin; STR: Streptomycin; NAL: Nalidixic; CIP: Ciprofloxacin; LEV: Levofloxacin; GEM: Gemifloxacin; AZI: Azithromycin; CT: Colistin; PB: Polymyxin B. *E. coli* ATCC 25922 was used as a control.

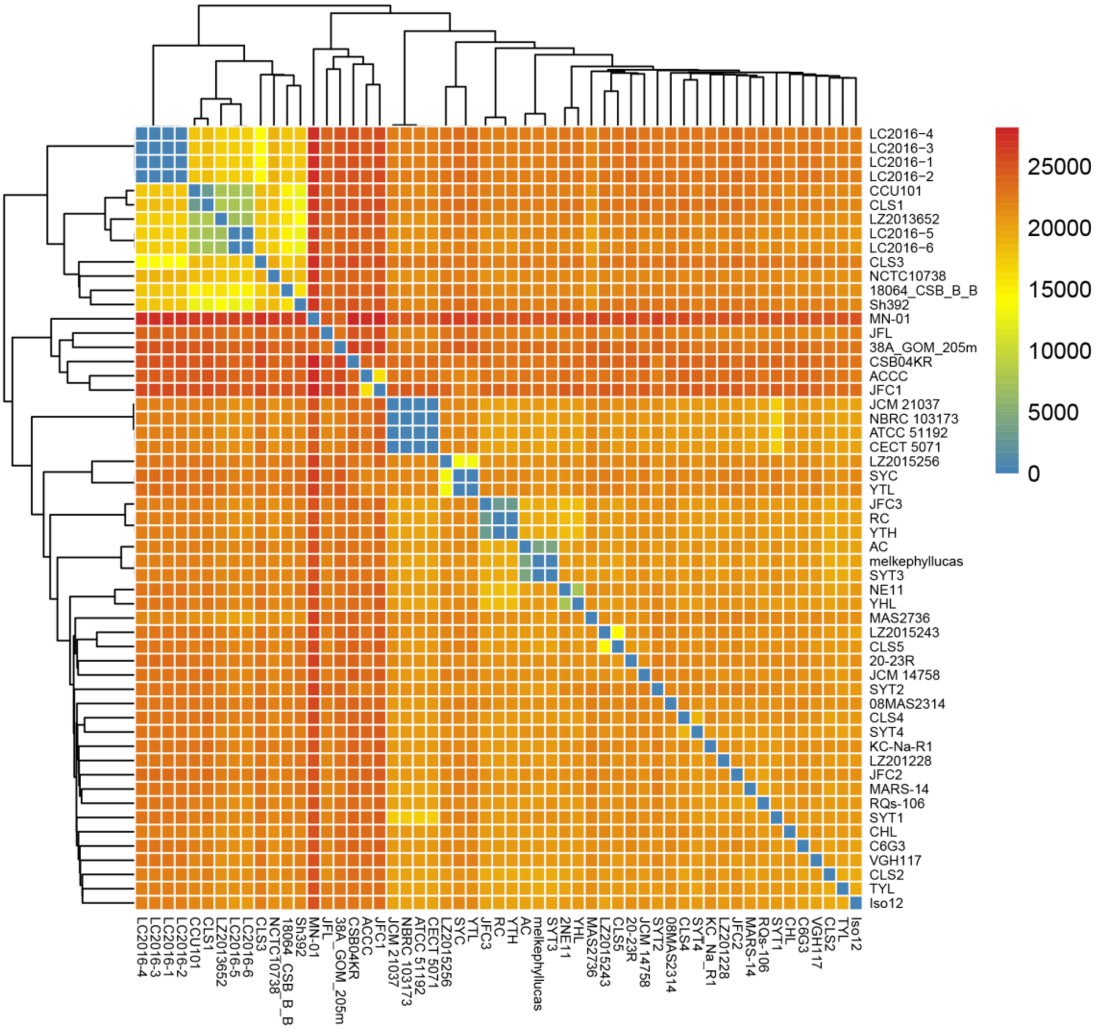


Figure S1 The distribution of pairwise SNP distances of 55 *S. algae* strains.

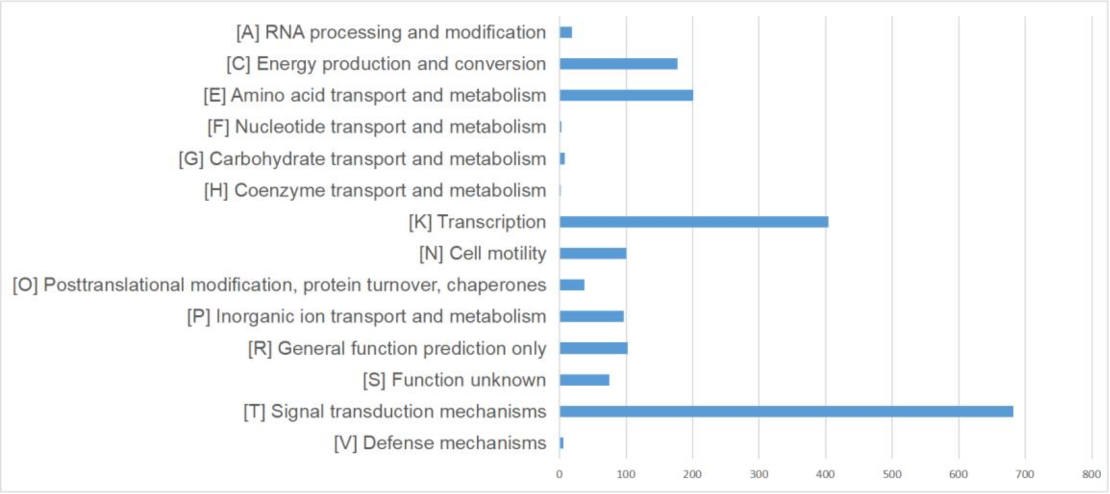


Figure S2 The COG functional annotation of the 55 strains unique genome.
